# New strategies to address world food security and elimination of malnutrition: future role of coarse cereals in human health

**DOI:** 10.3389/fpls.2023.1301445

**Published:** 2023-12-01

**Authors:** Xin Zou, Jieyu Zhang, Ting Cheng, Yangyang Guo, Li Zhang, Xiao Han, Changying Liu, Yan Wan, Xueling Ye, Xiaoning Cao, Chao Song, Gang Zhao, Dabing Xiang

**Affiliations:** ^1^Key Laboratory of Coarse Cereal Processing, Ministry of Agriculture and Rural Affairs, College of Food and Biological Engineering, Chengdu University, Chengdu, China; ^2^Center for Agricultural Genetic Resources Research, Shanxi Agricultural University, Taiyuan, China; ^3^Agricultural Genomics Institute at Shenzhen, Chinese Academy of Agricultural Sciences, Shenzhen, China

**Keywords:** coarse cereal, drought, yield, nutrient function, yield-enhancement strategy

## Abstract

As we face increasing challenges of world food security and malnutrition, coarse cereals are coming into favor as an important supplement to human staple foods due to their high nutritional value. In addition, their functional components, such as flavonoids and polyphenols, make them an important food source for healthy diets. However, we lack a systematic understanding of the importance of coarse cereals for world food security and nutritional goals. This review summarizes the worldwide cultivation and distribution of coarse cereals, indicating that the global area for coarse cereal cultivation is steadily increasing. This paper also focuses on the special adaptive mechanisms of coarse cereals to drought and discusses the strategies to improve coarse cereal crop yields from the perspective of agricultural production systems. The future possibilities, challenges, and opportunities for coarse cereal production are summarized in the face of food security challenges, and new ideas for world coarse cereal production are suggested.

## 1 Introduction

Coarse cereals are mainly grain and legume crops other than Rice (*Oryza sativa* L.), Maize(*Zea mays* L.), Potato(*Solanum tuberosum* L.), Wheat(*Triticum aestivum* L.), and Soybean(*Glycine max* (L.) Merr.), such as Buckwheat(*Fagopyrum esculentum*), quinoa(*Chenopodium quinoa Willd*.), Oats(*Avena sativa* L.), Peas(*Pisum sativum* L.), Mung beans(*Vigna radiata* (L.) R. Wilczek), Sorghum(*Sorghum bicolor* (L.) Moench), Barley(*Hordeum vulgare* L.) and Lentils(*Lens culinaris Medik.*), among others. These crops are mainly grown in semi-arid areas, and most of the time they can only rely on rain-fed agricultural systems, with almost no external input (Rai et al., 2008). Most of these grains contain unique nutritional and functionally active ingredients that make a significant therapeutic contribution to human immunity and the treatment of various chronic diseases, such as weight management, diabetes prevention, Cancer prevention, Cardiovascular diseases (CVD), etc (Kaur et al., 2014). Their secondary metabolites or phytochemicals have antioxidant properties and are rich in high-quality protein, dietary fiber, vitamins and various minerals (especially micronutrients) (Bouis, 2000). More importantly, some compounds are helpful to fight ischemic stroke, cardiovascular disease, cancer, obesity and type II diabetes (Jones et al., 2000; Jones, 2006). With the increasing awareness of health care, more attention is being paid to coarse cereals, with promising development of the coarse cereal industry (Fu et al., 2020).

Globally, the support of environment, society and economy cannot be separated from the food system (Metabolic, 2017). In particular, the current global epidemic situation is still severe, and coupled with unstable global geopolitical risks, global food security faces serious challenges. Coarse cereals generally have short growth cycles and are important and, in fact, irreplaceable in restructuring the cropping industry (Rai et al., 2008). Coarse cereals are also important for improving dietary structure and promoting nutritional health. As such, We can consider it they play an important role in eradicating hunger, ensuring food security, and improving the accessibility and affordability of healthy diets. Therefore, strengthening the production of coarse cereals is one of the most important strategies for ensuring world food security.

In the field of commercial food, the public acceptance of coarse cereals and related research and development and investment have been ignored. People are paying more and more attention to food security and nutrition. In addition, the current population growth needs more grain output as a stable backing. The development prospects of these miscellaneous grains will be better and better in rural or urban markets, poor or rich markets. Coarse cereal plants are very resistant and adaptable, especially in the face of drought, making their production and output more stable under today’s complex and changing natural conditions. In recent years, many countries have gradually adjusted their planting strategies with a consideration of these plants’ strong resistance to drought conditions, leading to a significant increase in harvesting area of coarse cereals. However, extreme climatic conditions are posing new challenges to the production of coarse cereals worldwide. To date, research on coarse cereals has rarely been reported and we lack an in-depth understanding and strategies related to the improvement of cultivation conditions and management. This paper therefore describes the importance of coarse cereals for world food security, summarizes systematic strategies to increase their production by analyzing the current status of their production management, and further lays out future challenges, possibilities and opportunities for coarse cereal production, while providing new ideas for coarse cereal production worldwide.

## 2 The importance of coarse cereals in world food security and human health

### 2.1 Coarse cereals as an important addition to world food security

**Figure 1:**
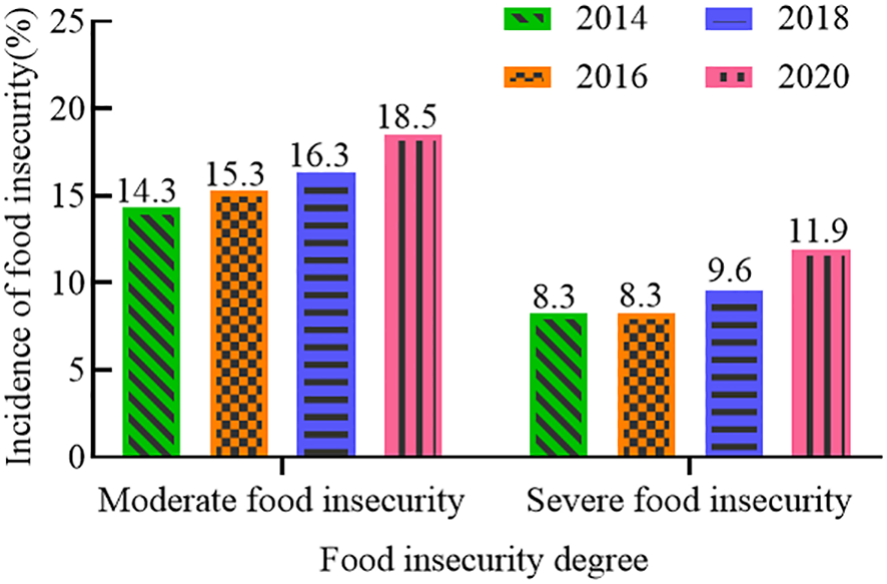
Incidence of food insecurity (%).

**Figure 2:**
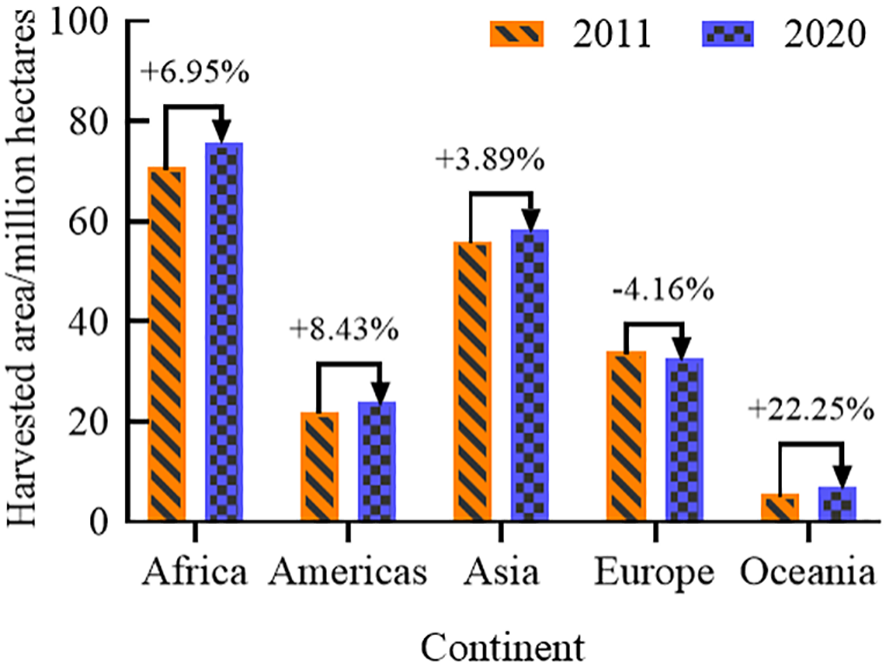
Distribution of harvested area of coarse cereals in global continents.

**Figure 3:**
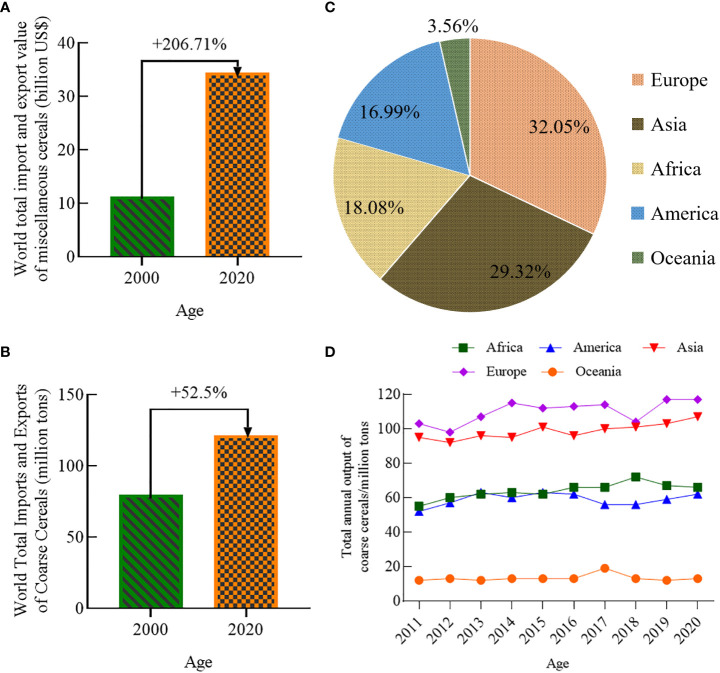
World total import and export value of coarse cereals (billion US dollars) **(A)**, World total imports and exports of coarse cereals (million tons) **(B)**, The proportion of total coarse cereal output on all continents in 2020 **(C)**, Total coarse cereal output on all continents from 2011 to 2020 **(D)**.

The 2022 Global Food Crisis Report, released by the Food and Agriculture Organization of the United Nations (FAO), indicated that the global food shortage in 2021 will have affected about 193 million people, involving a total of 53 countries or regions. In recent years, the global incidence of moderate to severe food insecurity has been on the rise (**Figure 1**) (FAO et al., 2022). From 2014 to 2020, the incidence of moderate food insecurity increased by 4.2%, and that of severe food insecurity by 3.6%. Since coarse cereals do not compete with major food crops for land, increasing their cultivation area has become a preferred option for many countries to increase production to meet market demand. The harvested area and production of coarse cereals (**Figures 2**, **3D**) worldwide are showing continuous increases, suggesting the potential for coarse cereals to eliminate hunger and ensure food security (Food and Agriculture Organization of the United Nations (FAO), 2022).

#### 2.1.1 Steady increase in the harvested area of coarse cereals

According to FAO, the global harvested area of coarse cereals was mainly distributed in semiarid and arid regions, showing a clear upward trend over 10 years (**Figure 2**). For example, from 2011 to 2020, a tremendous increase in the total harvested area of coarse cereals was noted in Oceania (+22.25%), followed by the Americas (+8.43%) and Africa (+6.95%). At the same time, in Europe, the total harvested area decreased by 4.16%.

**Figure 4:**
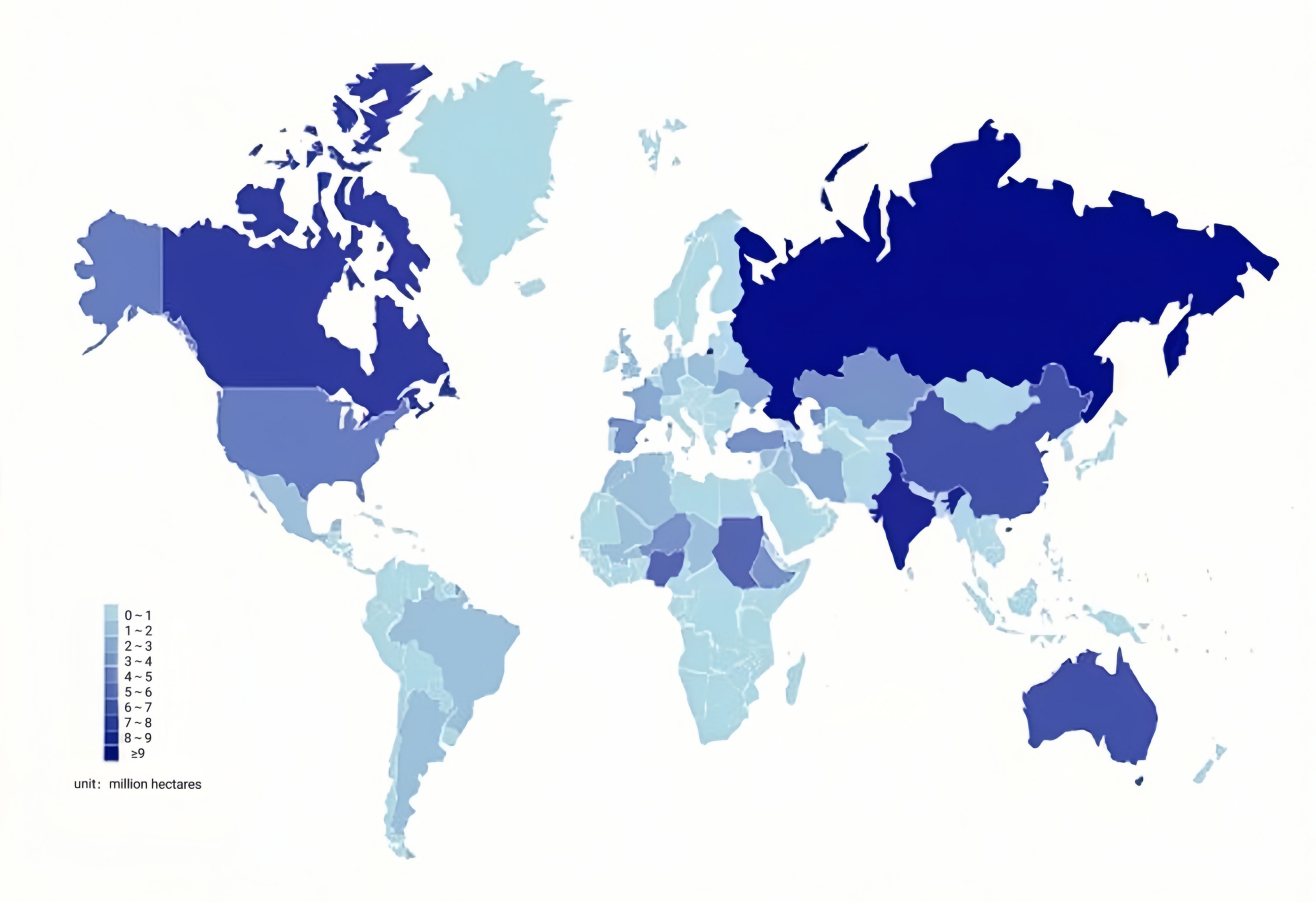
Distribution of coarse cereals in various countries in the world in 2020. Unit: million hectares.

The cultivation of different coarse cereals in different regions depends on the cultivation habits and climatic conditions. Therefore, the total harvest area of different coarse cereals in each country also varies greatly (**Figure 4**) (Food and Agriculture Organization of the United Nations (FAO), 2022). In 2020, many coarse cereals were grown in Russia, with the largest harvested area, including barley, buckwheat, and oats. These coarse cereals were also widely distributed in other countries. For instance, barley was distributed in Australia, Turkey, Canada, Spain, and the Republic of Kazakhstan. Buckwheat was distributed in China, and oats were grown in Canada, India, Australia, Spain, Poland, Brazil, the United States, Finland, and Argentina. Quinoa is mainly distributed in Bolivia, with the largest harvested area, as well as Peru and Ecuador; and sorghum is mainly distributed in Sudan, India, Nigeria, Niger, the United States, and Burkina Faso, with the largest area harvested in Sudan. Mung beans are mainly distributed in China, India, Thailand, Indonesia, and other countries, with the largest area harvested in China. Peas are mainly distributed in Canada, with the largest area harvested area, Russia, China, and India. Lentils are mainly distributed in Canada (largest area), India, and Australia. The total harvested area of coarse cereals in 2020 worldwide was 51.86 million hectares for barley, 2.25 million hectares for mung beans, 2.48 million hectares for buckwheat, 5.08 million hectares for lentils, 9.93 million hectares for oats, 8.13 million hectares for peas, 0.19 million hectares for quinoa, and 40.98 million hectares for sorghum.

#### 2.1.2 Steady increase in total production and trade of coarse cereals

The total world import and export value of coarse cereals increased from 11,244 to 34,487 billion US dollars from 2000 to 2020 (**Figure 3A**). The total world import and export volume of coarse cereals increased from 79.66 to 121.73 million tons (**Figure 3B**), an increase of 52.81%. Among the coarse cereals, barley, with a proportion of 58.19%, occupies an absolute dominant position in the import and export trade volume, while the trade volumes of quinoa and buckwheat are lowest, with a proportion of no more than 0.5%.

According to FAO (Food and Agriculture Organization of the United Nations (FAO), 2022), the share of total world coarse grain production in 2020 varied considerably between continents (**Figure 3C**); Europe and Asia were the main sources of coarse cereals, accounting for 32.05% and 29.32% of the world’s total production, respectively. Next, Africa and the Americas accounted for 18.08% and 16.99%, respectively, and Oceania accounted for the lowest proportion at 8.95%. From 2011 to 2020, the trend for the total global production of coarse cereals by continent followed a pronounced increase in Africa, the Americas, Asia, and Europe, while that in Oceania tended to remain the same (**Figure 3D**). The increased production in Africa, the Americas, and Asia was due to an increase in planted and harvested areas. In contrast, there was an increase in total production in Europe despite a decrease in harvested area, suggesting that the coarse cereal yield in the region increased, perhaps due to optimization of planting structures. Surprisingly, there was an increase in harvested area in Oceania; the fact that its total production remained the same might be due to the frequent occurrence of catastrophic climate events and changes in agricultural policies in Oceania in recent years.

Overall, the total global production and consumption of coarse cereals have gradually increased in recent years. The enhanced global yields of coarse cereals indicate increased investment in agricultural science and technology for these cereals. The closer attention paid to coarse cereals worldwide has promoted world trade and indicates that the international status of these crops has gradually risen with economic development.

### 2.2 Coarse cereals are important choices for human food security and a healthy diet

#### 2.2.1 Rich nutritional and functional components of coarse cereals

Unlike major food crops, coarse cereals are rich in nutritional components such as dietary fiber, amino acids, flavonoids, and phytosterols, functional components including polyphenols, glycols, anthraquinones, and alkaloids, and high-quality proteins, fats, and carbohydrates (Fu et al., 2020). The nutritional and functional components of different coarse cereals vary significantly, and are summarized in **Table 1** for barley, buckwheat, quinoa, oats, lentils, mung beans, sorghum, and peas, with more than 80 nutrients. Some of the functional components are very scarce, for example, chiral inositol, a sugar alcohol that is found only in buckwheat and mung beans (Hao et al., 2021). These nutritional functional components basically exist in all parts of the plants. In summary, the nutritional and functional components of coarse cereals are rich and diverse, providing more options for product processing and healthy dietary therapy.

**Table 1:** Nutrition and active compounds of coarse cereals.

Coarse cereals	Compounds	Original isolation	References
**Barley (*Hordeum vulgare* L.)**	Polyphenols, minerals, β-glucan, phenolic acids, flavonoids, tocopherols, phytosterols, folic acid	haulm, malt, seedling, seeds, sprouts, leaves, roots, fruits, grains, flowers, roots, stems, whole plants, flour	(Malik et al., 2014; Idehen et al., 2016; Sterna et al., 2017)
**Buckwheat (*Fagopyrum esculentum*)**	Rutin, kaempferol, quercetin glycosides, 2,6-dihydroxydaidzein, sissotrin, glycitin, genistin, ononin, catechin, epicatechin, epicatechin gallate, epigallocatechin, cyanidin glycosides, chiro-inositol	Seeds, sprouts, leaves, roots, fruits, grains, flowers, roots, stems, whole plants, flour	(Song et al., 2016; Sytar et al., 2018; Borovaya and Klykov, 2020)
**Quinoa (*Chenopodium quinoa Willd.*)**	Saponins, phytosterols, phytoestrogens, phenols, bioactive peptides, betaine, tannin, phytosteroids, flavonoids, γ-aminobutyric acid, kaempferol-O-dilauronosyl-galactosyl raw sugar, quercetin-O-glucuronide, ferulic acid, rutin, tocopherol	Seeds, sprouts, leaves, roots, fruits, grains, flowers, roots, stems, whole plants, flour	(Paucar-Menacho et al., 2018; del Hierro et al., 2020; Khan and Javaid, 2022; Villacrés et al., 2022)
**Oats (*Avena sativa* L.)**	β-glucan, avantamide, linoleic acid, kaempferol-3-O-galactoside-6’’-rhamnocitin-3’’-yun essence, hespeRetin-7-O-neoorange glycoside, ginsenoside re, soybean saponin ba, gallic acid, vanilloid, caffeic acid, syringic acid, p-coumaric acid, chlorogenic acid, ferulic acid	Seeds, sprouts, leaves, roots, fruits, grains, flowers, roots, stems, whole plants, flour	(Jiang et al., 2021; Klajn et al., 2021; Wei et al., 2021)
Lentils (*Lens culinaris Medik.*)	Dietary fiber, arabinoxylan, single phenolic compounds, tocopherols, catechins, kaempferol, quercetin derivatives, saponins, phytosterols, lectins, defensins, protease inhibitors, resistant starch, oligosaccharides, bioactive peptides, dietary fiber, minerals, antioxidants, vitamins, flavonoids, polyphenols, phenolic acids	Seeds, sprouts, leaves, roots, fruits, grains, flowers, roots, stems, whole plants, flour	(Menga et al., 2014; Shahwar et al., 2017; Ciudad-Mulero et al., 2018; Herrera et al., 2019; Galgano et al., 2021)
**Mung beans (*Vigna radiata* (L.) R. Wilczek)**	Polyphenol, polysaccharide, peptide, gamma-aminobutyric acid, flavonoid, tocopherol, glycine, neochlorogenic acid, chlorogenic acid, vitexin, isovitexin, caffeic acid, catechin, syringic acid, p-coumaric acid, chiro-inositol	Seeds, sprouts, leaves, roots, fruits, grains, flowers, roots, stems, Whole plants, flour	(Landete et al., 2015; Xue et al., 2016; Hou et al., 2019; Lim et al., 2022; Ma et al., 2022)
**Sorghum (*Sorghum bicolor* (L.) Moench)**	Carotenoid, vitamin E, amines, phytosterol, ranunculin, glycine, ononin, anthocyanins, flavonoids, phenolic acids, stilbene, tannin, phenol, quinine formate, quinine dihydrochloride, chloroquine	Seeds, sprouts, leaves, roots, fruits, grains, flowers, roots, stems, Whole plants, flour	(Coulibaly et al., 2020; Przybylska-Balcerek et al., 2020; Li et al., 2021; Bianco-Gomes et al., 2022; Khalid et al., 2022)
**Peas (*Pisum sativum* L.)**	Anthocyanin, vitamin c, phenol, flavonoid, carotenoid, 5-caffeoyl quinic acid, epicatechin, hesperidin, condensed tannin, rosmarinic acid, rutin, galangin, moline, naringin, hesperetin, pinosson protein, flavonol glucoside	Seeds, sprouts, leaves, roots, fruits, grains, flowers, roots, stems, Whole plants, flour	(Menga et al., 2014; Saberi et al., 2018; Salacheep et al., 2020; Castaldo et al., 2022)

#### 2.2.2 The important role of functional components in coarse cereals

Studies show that all coarse cereals contain functionally active ingredients that improve immunity and can be used to treat chronic diseases. Hostetler et al. (2017) summarized the polyphenolic components (flavonoids and phenolic acids) that are enriched in coarse cereals, which exhibit antioxidant properties by scavenging or reducing free radicals in the body. Polyphenolic components also have hypoglycemic, lipid-lowering and anticancer effects (Anantharaju et al., 2016). Anthraquinones, present in coarse cereals, are commonly used in laxatives because of their strong activity (Malik and Muller, 2016). Zou et al. (2021) concluded that buckwheat is a medicinal food crop, and its functional components are effective at reducing the incidence of tumors, atherosclerotic cardiovascular diseases, hypertension, and diabetes. β-Glucan, which is highly enriched in oats, can improve immune function by enhancing metabolism and modulating immune cell responses (Ji et al., 2003). Vitamin E, which is rich in barley, prevents aging, protects the skin, and is also effective at promoting blood circulation (Do et al., 2015).**Table 2** shows all the compounds found only in coarse cereals and their importance to health.

**Table 2:** Unique functional components in coarse cereals and their importance to health.

Component	Properties	References
**Fagopyrins**	Laxative, antibiotic, antiviral effects, treatment for diabetes	(Hagels, 2007)
**Fagopyritols**	Against type II diabetes, polycystic ovaries development, and plasma cholesterol	(Khalid et al., 2020)
**Rutin**	Higher antioxidant, anti-inflammation, anticancer, hepatoprotective activity, neuroprotective effects	(Choi et al., 2015; Agnihotri and Aruoma, 2020; Ruan et al., 2020)
**Avenanthramides**	Anti-inflammatory, anti-atherogenic, antioxidant	(Emmons et al., 1999; Chen et al., 2004)
**d-fagomine**	Against diabetes, pathogenic diseases, cancer, AIDS, overweight, and viral diseases	(Amezqueta et al., 2012)
**avenanthramides**	Antioxidant, anti-inflammatory, anti-colon cancer, vasodilation, antipruritic, cytoprotection, cholesterol lowering	(Katz et al., 2001; Saltzman et al., 2001; Ji et al., 2003; Liu et al., 2004; O’Moore et al., 2005; Kurtz and Wallo, 2007; Guo et al., 2010; Xue et al., 2021)

#### 2.2.3 The relationship between coarse cereals and a healthy diet

According to United Nations International Children’s Emergency Fund (FAO et al., 2022), the challenges to achieving the 2030 global nutrition goals remain enormous; in 2020, an estimated 149 million (22%) children (under 5 years of age) worldwide exhibited stunted growth and 45.4 million (6.7%) children under 5 showed signs of wasting. At the same time, the global cost of healthy diets has increased significantly, and therefore their affordability has decreased. In 2020, the population who were not able to afford healthy diets was approximately 3.1 billion, 112 million more than in 2019. In addition, surveys by FAO (FAO et al., 2022) suggest that the economic impact of COVID-19 and the measures taken to contain it have led to higher consumer costs for food. However, the reality may be even bleaker, because the impact of the COVID-19 outbreak on human nutritional indicators is still under observation.

In conclusion, coarse cereals have become one of the major food sources for human health due to their dual roles as functional foods and healthy dietary supplements. The United States Food and Drug Administration (FDA) recommends a daily intake of more than 3 g of β-glucan soluble fiber, which can reduce cholesterol by 23%, and cereals containing β-glucan are classified as functional foods (Bernstein et al., 2013; Maheshwari et al., 2019). With the increasing awareness of coarse cereals and their nutritional and functional benefits, more research on coarse grains and their application to dietary guidelines are expected.

## 3 Response mechanisms in coarse cereals for drought adaptation

Arid and semiarid areas account for about 35% of the world’s land mass, with a yearly increasing tendency. The reduction in crop yield caused by drought exceeds the total yield reduction caused by other environmental factors (Xiang et al., 2020). Approximately 25% of global agricultural land is affected by drought (Hidri et al., 2016), making it one of the major abiotic factors limiting crop production. Coarse cereals have a range of morphological and physiological responses to different environments. The long-term cultivation of coarse cereals could promote their adaption to drought conditions by regulating their morphological and physiological responses to drought (Sayed et al., 2012; Nxele et al., 2017; Lin and Chao, 2021).

### 3.1 Distribution of coarse cereals in relation to climate

**Figure 5:**
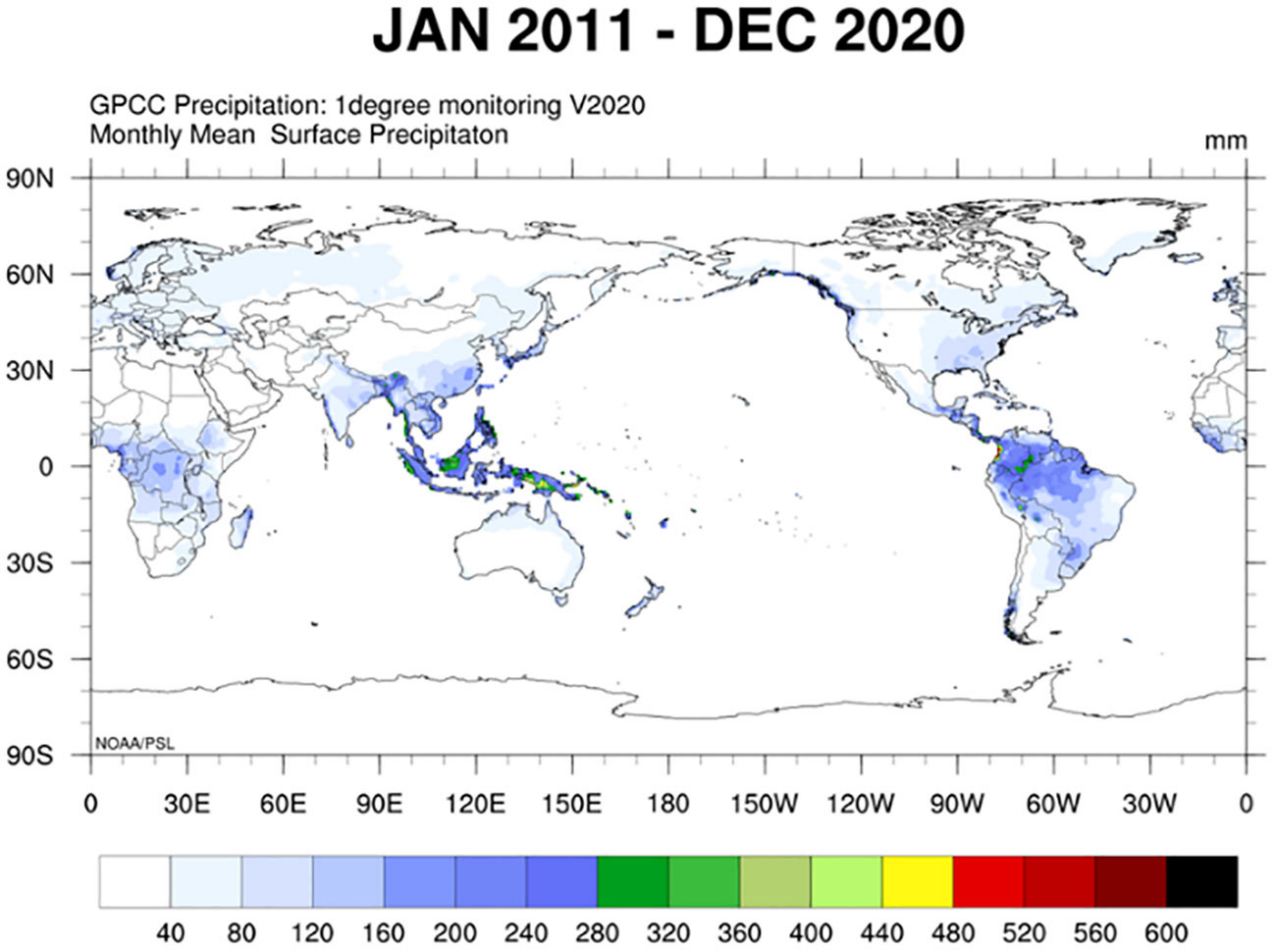
The global average monthly surface precipitation (From January 2011 to December 2020). Unit: mm.

**Figure 6:**
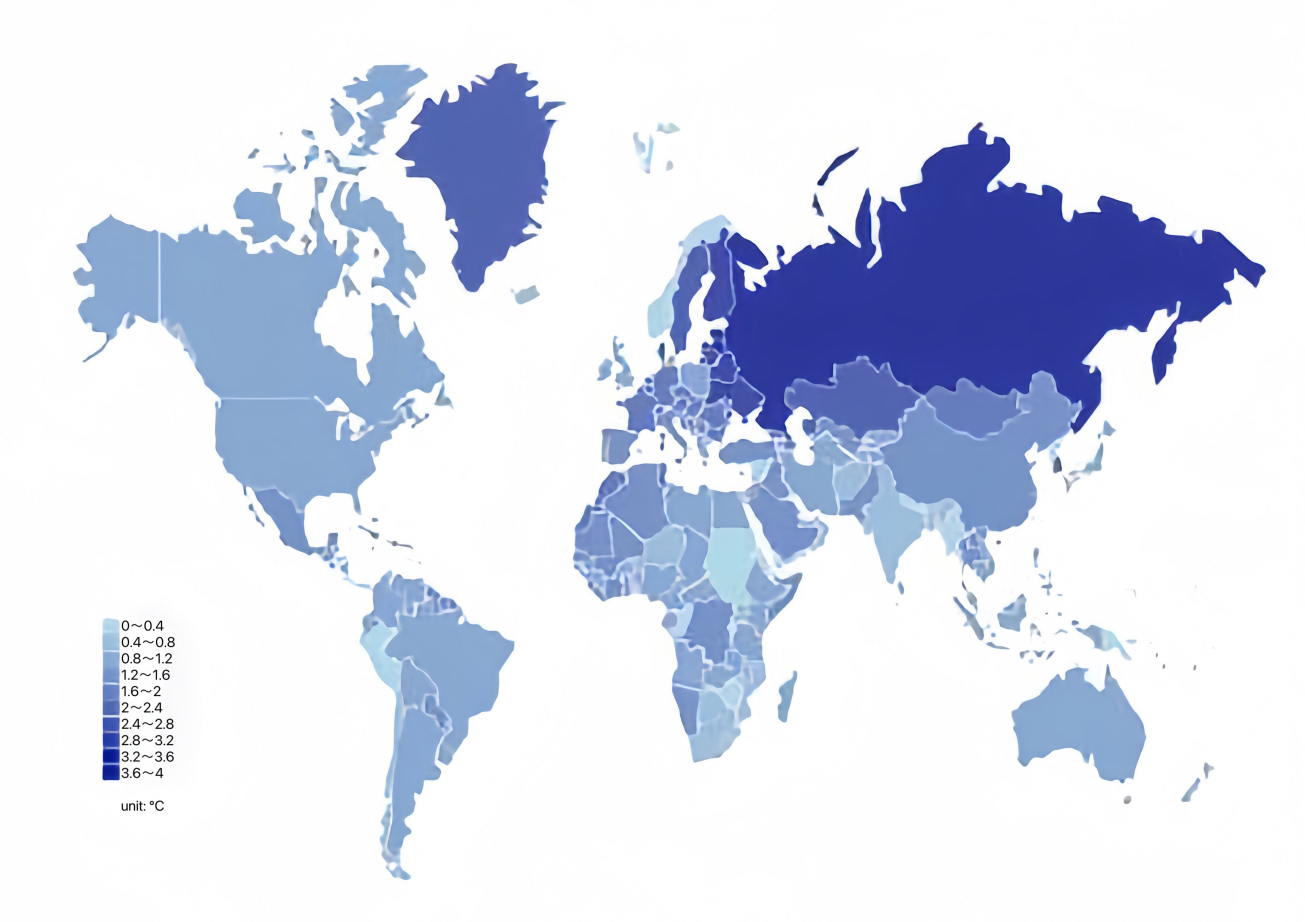
Global temperature change in 2020. Unit: °.

By simultaneously observing the global distribution and production of coarse cereals (**Figures 4**, **3**) and global changes in precipitation and temperature by country or region (**Figures 5**, **6**), we found that the harvested area, temperature changes, and total coarse cereal production show a simultaneous increase, while the change in monthly average surface precipitation presents an opposite trend. These trends illustrate that countries or regions with water scarcity and aridity have larger harvested areas of coarse cereals. In contrast, countries or regions with high rainfall have relatively lower harvested areas and total production of coarse cereals. This may be because the low precipitation conditions are unsuitable for other major food crops, whereas coarse cereals adapt well to water deficiency, can be grown in more areas and produce better yields, making them a better choice for arid or semiarid regions (Killi and Haworth, 2017).

### 3.2 Morphological and physiological response mechanisms for drought adaptation in coarse cereals

The required mechanisms to cope with drought in mixed crops are complex and synergistic. The crop responds to water deficit by reducing respiration, increasing the activity of its own antioxidant enzymes and non-enzymatic antioxidant content, and increasing the content of osmoregulatory substances to improve cell water retention.

#### 3.2.1 Growth period and morphological adaptations

We found that most coarse cereals can enhance their adaptability to the environment by altering their growth period or morphology. **Table 3** summarizes the fertility period of different coarse cereals. The average fertility period of coarse cereals is around 58-62 days shorter than that of the major food crops. At the same time, coarse cereals adapt to the environment through certain morphological changes under drought conditions. Sallam et al. (2019) found that barley reduces plant height and leaf number under the condition of water shortage, as compared to controls, and the thickness of the leaf tissue and diameter of the vascular bundle in flag leaves are correspondingly reduced to adapt to the drought conditions. Lentils adapt to drought conditions by leaf drying, leaf curling and slowed growth rate, thereby reducing water and nutrient consumption (Akter et al., 2021). Buckwheat flowers and sets early under drought conditions, with rapid leaf decline and a shortened reproductive period (Hou et al., 2019).

**Table 3:** Nutrition and active compounds of coarse cereals.

Item	Crops	Growth period (d)	References
**Main Crops**	Rice	107-135	(Cheng et al., 2018)
	Maize	125-131	(He et al., 2020)
	Soybean	91-165	(Tan et al., 2021)
	Wheat	242-246	(Fujiwara et al., 2010)
**Mean**	/	142-170	/
**Coarse cereals**	Barley	80-106	(Hakala et al., 2020)
	Buckwheat	56-60	(Jung et al., 2015)
	Quinoa	107-158	(Tan and Temel, 2018)
	Oats	83-110	(Jung et al., 2015)
	Lentils	126-130	(Singh O. et al., 2022)
	Mung beans	70-90	(He et al., 2022)
	Sorghum	90-110	(Raymundo et al., 2021)
	Peas	60-100	(Garmendia et al., 2022)
**Mean**	/	84-108	/

"/" indicates that there is no content here.

#### 3.2.2 Physiological response

**Figure 7:**
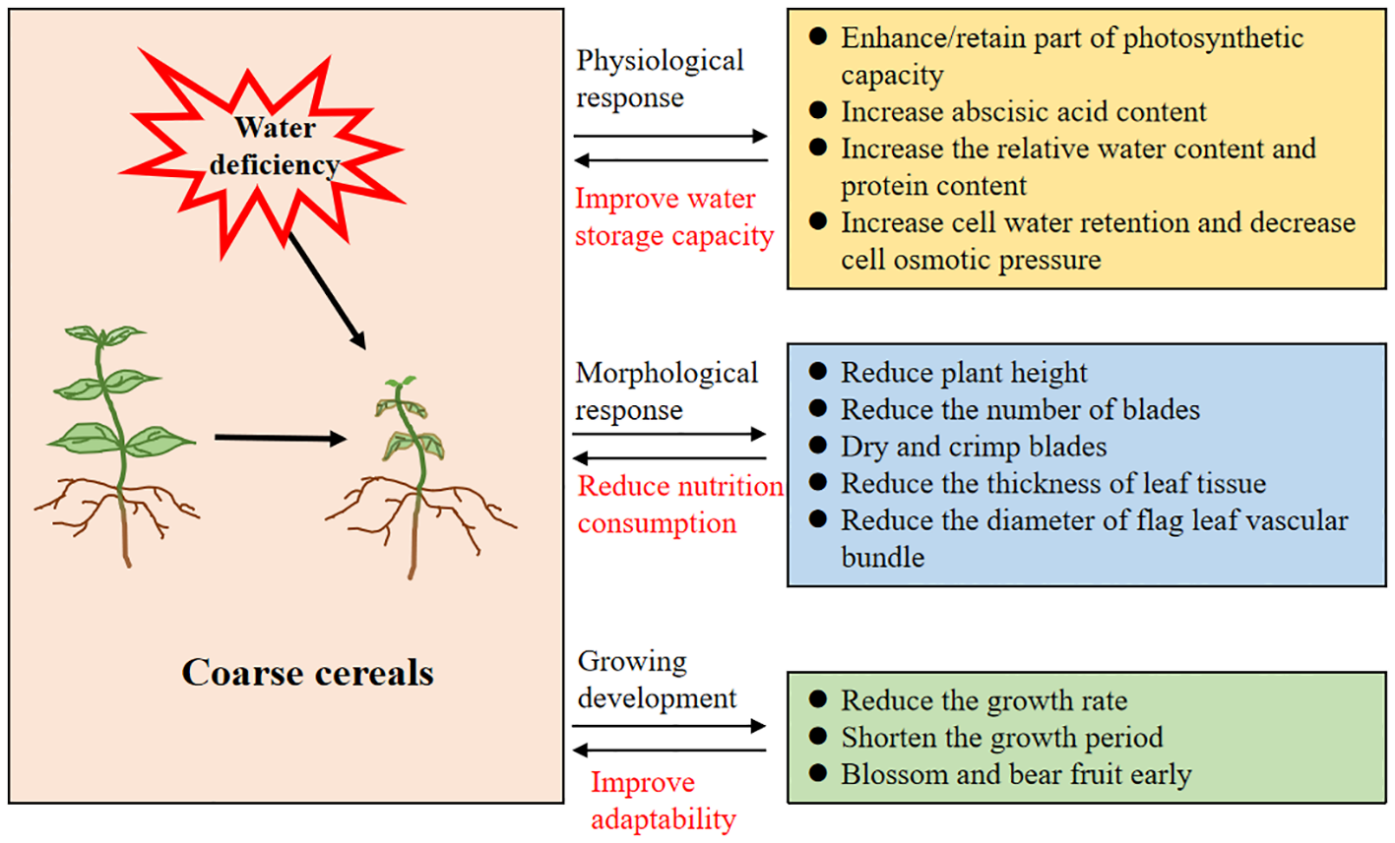
Main ways of drought stress inhibiting crop growth.

As shown in **Figure 7**, water deficiency causes leaf curling and wilting, and photosynthesis inhibition, resulting in reduced crop growth. In addition, coarse cereals develop rapid physiological responses or specific adaptive mechanisms under drought conditions, making them more drought-tolerant or drought-resistant. For instance, drought limits the operation and metabolism of photosynthesis in quinoa, but the plant still retains some of its photosynthetic capacity to maintain its growth and development; thus, it is strongly drought-tolerant (Killi and Haworth, 2017). Moreover, its photosystem II is better adapted to drought and has a relatively more efficient photosynthetic capacity during the nutritional phase compared to other growth stages (Fghire et al., 2015). Under drought conditions, coarse cereals such as buckwheat can accumulate more proline to cope with the adverse effects of water shortage (Yao et al., 2017). Certainly, the rapid response and changes in hormone levels are also important regulatory means, and the rapid increase in abscisic acid content in barley leaves under water deficit inhibits leaf transpiration and water transport in the root system; thus, early maturation of barley is induced without altering grain composition—proper grain development is ensured and maintained (Staroske et al., 2016). *In vivo*, 1-aminocyclopropane-1-carboxylate deaminase (ACC deaminase) mitigates the negative effects of water deficiency by reducing ethylene levels and increasing relative water content of lentils (Zafar-ul-Hye et al., 2021). Similarly, mung beans respond rapidly to drought at the hormonal level, exhibiting promoted growth via inhibition of ethylene synthesis (Sarapat et al., 2020).

Osmolytes are low-molecular-weight organic compounds or compatible highly soluble solutes, including amino acids, sugars, sugar alcohols, and quaternary ammonium compounds (Bilal et al., 2019). In addition to hormonal alterations, when confronted with a water-deficient environment, coarse cereals can quickly adapt to the conditions and satisfy their growth needs by completing osmoregulation of osmolytes at the cellular level. Under drought conditions, coarse cereals tend to increase the concentration of osmolytes, such as proline, inositol, alginates, and betaine, in order to reduce cellular osmotic pressure and maintain cellular water retention (Khan et al., 2015).

At present, some drought-induced TFs have been identified (FtMYB9,FtMYB10,FtMYB13,from FtNAC2 to FtNAC9,FtbZIP5 and FtbZIP83,FtbHLH3), But it mainly focuses on the changes of gene regulation of tartary buckwheat under drought (Gao et al., 2016; Gao et al., 2017; Huang et al., 2017; Yao et al., 2017; Deng et al., 2019; Li et al., 2019; Sun et al., 2019).Many studies have shown that the gene encoding LEA protein is induced to maintain the stability of membrane and protein, and can alleviate cell damage under water shortage conditions (Shinozaki and Yamaguchi-Shinozaki, 2007; Roychoudhury et al., 2013; Shi et al., 2020). The continuing conundrum of the LEA proteins. Naturwissenschaften 94:791-812.Huang et al. conducted the transcriptome analysis of tartary buckwheat under drought stress for the first time, and confirmed that LEA protein participated in the response of tartary buckwheat to drought stress (Huang et al., 2021). Development and flowering time of barley were correlated with a differential down-regulation of the flowering promoters s flowering locus T1 and the Barley mads-box genes BM3 and BM8. The researchers also found that that PPD-H1 Affects developmental plasticity in response to distress in Barley (Gol et al., 2021). Researchers discovered gene-LOC110713661 and gene-LOC110738152 May be the key genes for troubleshooting tolerance in quinoa through transcript ome and metablome association analysis (Huan et al., 2022).The expression levels of CqCIPK11, CqCIPK15, CqCIPK37 and CqCBL13 increased significantly under drought stress (Zhu et al., 2022). On the other hand, the up regulated of PP2C, ABF, SNR K2, GID1, Jaz, and MyC2 genes may enhance the fault tolerance of oat (Gong et al., 2022). It is confirmed by experiments that drought will cause high expression of VrNAC13, so researchers speculate that this gene can regulate the stress resistance of mung beans (Zhang et al., 2023). SbNAC9 improves drought tolerance by enhancing scavenging ability of reactive oxygen species and activating stress-responsive genes of sorghum (Zheng et al., 2023).

### 3.3 Synergistic effects of drought and specific functional components of coarse cereals

The regulation of secondary metabolites has also been found to be associated with drought (Hassan and Basahi, 2014). Drought can promote the formation of secondary metabolites in coarse cereals, which in turn might be involved in regulating the coarse cereals’ own defense against unfavorable external environmental conditions, allowing for the maintenance of normal growth and development. In the process of long-term adaptation to adversity, coarse cereals have formed a synergistic relationship between adverse conditions and qualitative changes: usually, plants increase the biosynthesis of functionally active components, such as phenolic compounds, flavonoids, and anthocyanins, when exposed to drought conditions (Zahedi et al., 2021). It was found that secondary metabolites in barley may act as antioxidants, regulators of gene expression, and regulators of protein function during conditions of water deficit (Piasecka et al., 2017). Researchers have also found that drought significantly increases the total flavonoid content in sorghum (Kamali and Mehraban, 2020). Total flavonoid and rutin contents in buckwheat are also significantly increased under drought conditions (Meng et al., 2022). Flavonoids are secondary metabolites with high antioxidant activity; they scavenge reactive oxygen radicals, which in turn attenuates the damage caused by reactive oxygen radicals in buckwheat (Olmo et al., 2014). Unfortunately, the relationship between the production of many secondary metabolites and drought or other adverse conditions is still unknown. More research is needed to determine the roles and feedback mechanisms of secondary metabolites in the drought response.

## 4 Management strategies for enhancing coarse cereal yields

**Figure 8:**
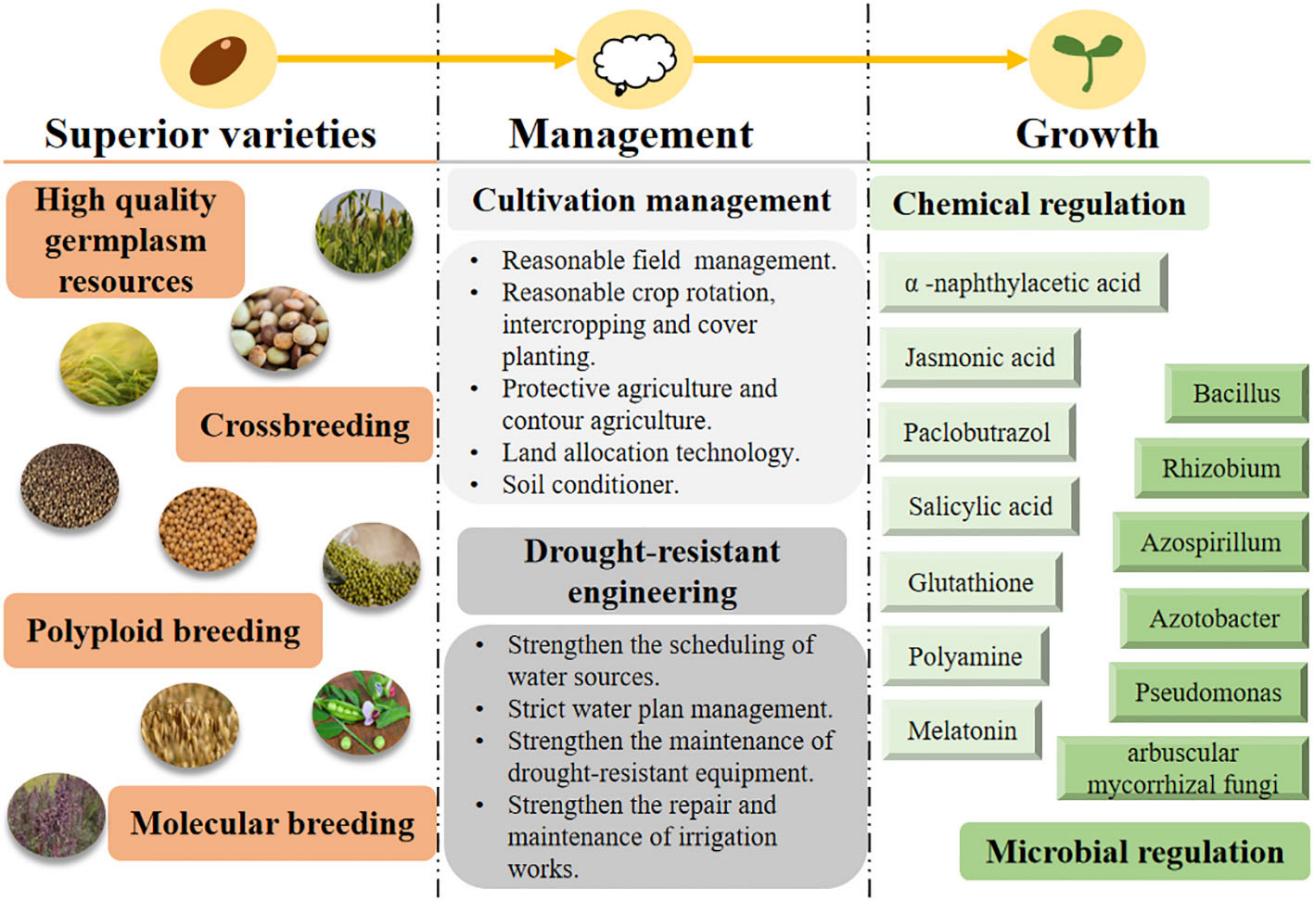
Management strategy for increasing production of coarse cereals in arid areas. AMF, arbuscular mycorrhizal fungi.

In view of the described characteristics of coarse cereals, this paper summarizes management strategies aimed at increasing the yield of coarse cereals in dry areas (**Figure 8**). Based on the high yield and high efficiency of these cereals, we suggest that the following five aspects be taken into consideration.

### 4.1 Optimal seed breeding and application

Seeds are the core of agriculture and play an important role in the harvest of high-quality, high-yielding coarse cereals (Xiang et al., 2016; Xiang et al., 2019a). In arid and semiarid regions, there is an urgent need to select and breed drought-tolerant crop varieties and lines that can produce high yields (Seleiman et al., 2021). Therefore, germplasm resources with different characteristics must be collected and mined for superior genes. The following traits have been suggested as key indicators of drought-tolerant crops: short growth cycle (Mwamahonje et al., 2021), high root density (Kothari et al., 2020), high osmoregulatory activity in roots (Haling et al., 2013), high stomatal conductance (Leybourne et al., 2022), high leaf relative water content (RWC) (Yamamoto et al., 2000), optimal water utilization (Guenther et al., 2003), and high plasticity (Gano et al., 2021).

### 4.2 Proper management practices in field cultivation

One important approach to enhancing drought resistance in coarse cereals is to optimize the sowing time, planting density, and field management for the climatic and environmental conditions (**Table 4**). Proper crop rotation, intercropping, and cover cropping can effectively increase the content of soil organic matter (Turmel et al., 2011), improve soil physicochemical properties (Lehman et al., 2012), and increase soil fertility (Salehi et al., 2018), by making full use of space and other resources (Araya et al., 2021). These measures can improve light-transmission conditions, increasing the efficiency of light-energy utilization in coarse cereals (Flower et al., 2012; Xiang et al., 2019b) and contributing to increased yield.

**Table 4:** Soil environment and planting measures suitable for coarse cereals.

coarse cereals	buckwheat	quinoa	oats	peas	mung beans	sorghum	barley	lentils
**soil types**	loam	loam	sandy loam	loam	Sand, loam, clay	silt loam, sandy loam, clay	sandy loam	sandy loam
**Soil pH**	weakly acidic-neutral	weakly acidic-weakly basic	weakly acidic-neutral	weakly acidic-neutral	neutral	weakly acidic-weakly basic	neutral	Neutral-weakly basic
**Planting mode**	rotate crops	rotate crops	rotate crops	rotate crops	rotate crops	rotate crops	rotate crops	rotate crops
**Sowing date**	mid-April	mid-April *vs*. early May	early March *vs* early May	March to May	early April	May to June	late September *vs* early October	late April mid-April
**Main diseases**	damping-off, leaf spot	downy mildew, root rot	Smut, crown rust, stem rust	powdery mildew, uromyces viciae-fabae	Leaf spot, anthracnose, rust	anthracnose	scab, powdery	wilting, root rot
**Main pests**	aphid	eurysacca melanocampta meyrick	aphid	aphid	pod borer	aphid, chilo partellus	aphids	pea leaf weevil
**references**	(Jonsson et al., 2009; Wang et al., 2019; Song et al., 2020; Wu et al., 2020; Shen et al., 2021; Erol et al., 2022; Zini et al., 2022)	(Costa et al., 2009; Tekin et al., 2017; Kandel et al., 2019; Adamczewska-Sowinska et al., 2021; Colque-Little et al., 2021; Domingos and Bilsborrow, 2022)	(Zhao et al., 2010; Ferrazza et al., 2013; Benevenuto et al., 2018; Delin et al., 2018; Kebede et al., 2019; Umina et al., 2020; Jordan-Meille et al., 2021; Li et al., 2022)	(Azpilicueta et al., 2012; Fazalova and Nevado, 2020; Abbas et al., 2022; Beniwal et al., 2022; Woo et al., 2022)	(Shen et al., 2010; Mehmood et al., 2018; Indiati et al., 2021; Hussain et al., 2022; Mott et al., 2022; Tariq et al., 2022)	(Holou and Stevens, 2012; Singh et al., 2012; Silva et al., 2015; Felderhoff et al., 2016; Oten, 2017; Punnuri et al., 2022)	(Chamanabad et al., 2009; Tripolskaja et al., 2013; Ames et al., 2015; Mirosavljevic et al., 2015; Benevenuto et al., 2018; McKee et al., 2019; Escudero-Martinez et al., 2020; Jordan-Meille et al., 2021)	(Akhtar et al., 2010; Reddy et al., 2018; Pala, 2019; Bazghaleh et al., 2020; Maphosa et al., 2022; Singh M. et al., 2022)

Conservation tillage, which prevents soil erosion and land-quality degradation (Roohi et al., 2022) is important for the construction of sustainable agricultural production systems for coarse cereals. One conservation tillage method consists of applying soil amendments, including compost (Hinojosa et al., 2018), chitosan (Hafez et al., 2020), and vermicompost (Benaffari et al., 2022), which play important roles in promoting the growth of coarse cereals and their resistance to drought. Other soil amendments, such as biochar, can improve the soil’s water-holding capacity and reduce ion and infiltration toxicity (Thomas et al., 2013). Enzyme activities in the improved soil and soil water-use efficiency will also increase (Atkinson et al., 2010; Wang et al., 2014). It has also been suggested that biochar combined with alternate root-zone drying irrigation may be a sensible way to maintain crop productivity in arid and semiarid regions of the world, thereby ensuring food security (Yang et al., 2020).

Chemical regulation is a rapid and easy growth-regulating measure in the production of coarse cereals; it is an indispensable tool for improving morpho-physiological processes in these cereals. Application of exogenous melatonin enhances antioxidant enzyme activity, reduces oxidative damage, and improves photosynthetic capacity and drought tolerance in buckwheat (Hossain et al., 2020) and oats (Gao et al., 2018). Foliar application of polyamides on mung bean improved yield by reducing the damage caused by water deficiency (Babarashi et al., 2021). In addition, exogenous application of polyamines on mung bean also increased the photosynthetic rate and induced the accumulation of osmoprotectants, improving its tolerance to water deficits (Sadeghipour, 2019). Foliar sprays of jasmonic acid on quinoa were found to increase yield by 16% compared to controls under water-deficit conditions (Keshtkar et al., 2022). Canales et al. (2019) found that salicylic acid can interact with polyamines in a complex way to build a signaling network that mitigates the adverse effects of water deficiency on oats (Gong et al., 2022). In addition to these chemicals, exogenous supplementation of glutathione during droughts can significantly enhance antioxidant components, which in turn reduce oxidative damage, indicating its significant role in improving drought resistance (Nahar et al., 2015).

### 4.3 Microbial regulation in root development and soil environment

Microbial regulation can promote plant adaptation to stressful conditions. Microorganisms secrete viscous and water-absorbing macromolecules into plant roots during proliferation (Marulanda et al., 2009), stimulate plant growth (Miransari, 2014) and reduce transpiration of water from soil capillary pores (Augé, 2001), inducing soil insulation, warming, and soil-moisture retention. Under water-deficit conditions, microorganisms can alleviate the adverse effects of drought on crops, promote crop growth and development, improve nutrient- and water-uptake efficiency, and increase yield (Bolan, 1991; Karagiannidis and Hadjisavva-Zinoviadi, 1998; Liu et al., 2000). Qiao et al. (2011) was the first to report on the enhancement of drought tolerance by arbuscular mycorrhiza colonization in peas. It was also found that plant growth-promoting rhizobacteria combined with arbuscular mycorrhizal fungi(AMF) improve drought tolerance in peas and lentils (Figueiredo et al., 2008).

### 4.4 Water-saving production systems

Water-saving production systems are an important strategy for coarse cereals, and for future development directions toward increasing their yields in dry areas. On the one hand, these systems can greatly improve the efficiency of limited water resource utilization, and on the other, they can facilitate the realization of increased yield of coarse cereals in dry zones. Unfortunately, irrigation water-saving systems are currently lacking in dryland cultivation of coarse cereals (Levidow et al., 2014). Therefore, the task of exploring reasonable engineering measures for drought resistance in coarse cereals is daunting. According to Du et al. (2015), a fully integrated system of sustainable water allocation is required, and deficit irrigation can be used rather than improved agricultural water allocation. Deficit irrigation plays a tremendous role in controlling the water use of crops at different stages of cultivation and in actively regulating plant growth, productivity, and development according to the crop’s physiological response.

## 5 Conclusion and perspectives

**Figure 9:**
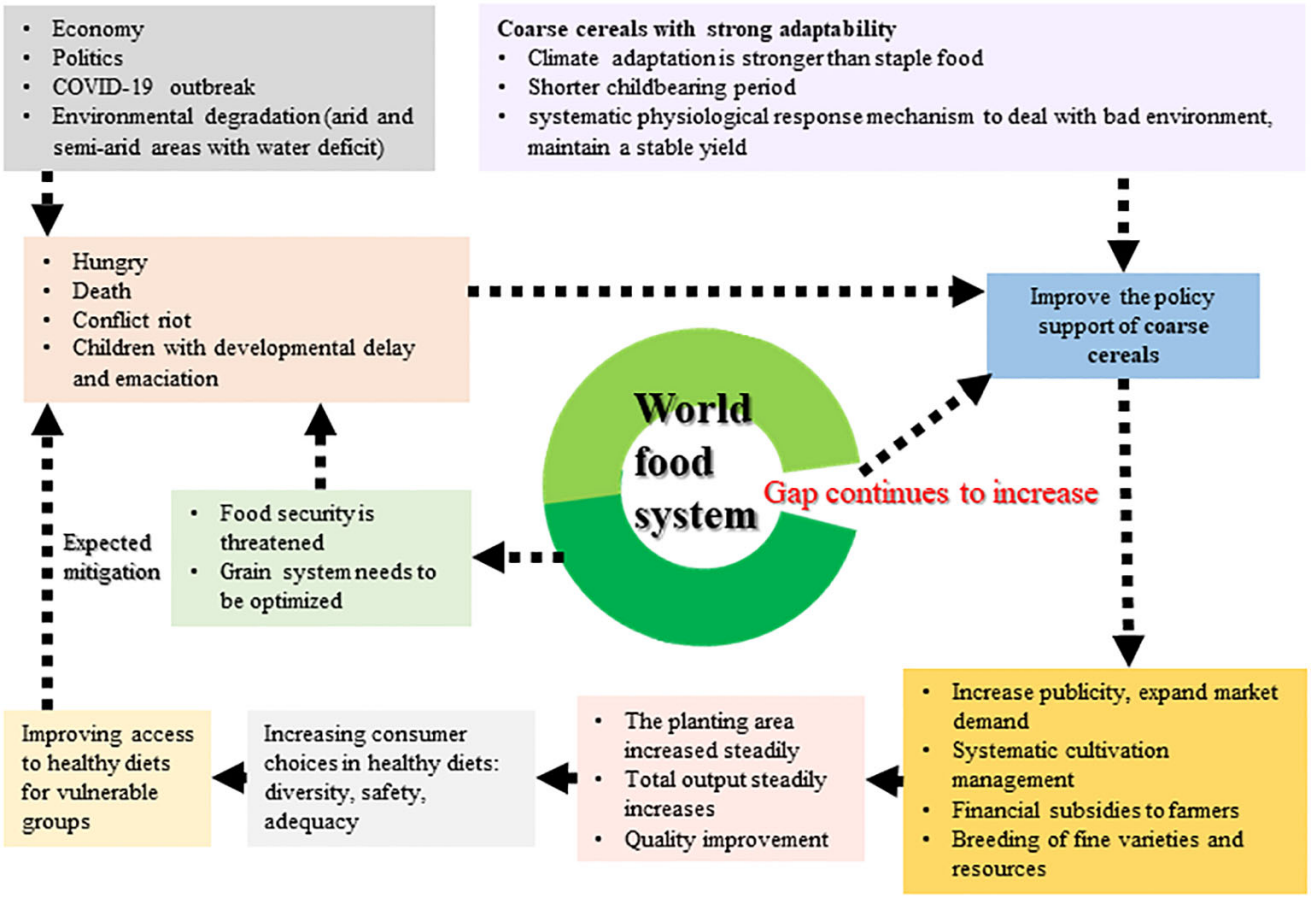
Systemic solution of coarse cereals based on. First, policy support for crude processing of coarse cereals should be increased, followed by more publicity to expand market demand, as well as systematic cultivation management and selection and breeding of good varieties, without forgetting financial subsidies for farmers. Following these steps will result in a steady increase in planting area and production, and improved quality of agricultural products. The resultant wide variety of coarse cereals will increase consumers’ choices for a healthy diet. In addition, the low price of coarse cereals can facilitate access to healthy diets for vulnerable groups.

Based on the complicated and changeable situation in the world today, this review draws up a systematic solution for coarse cereals (**Figure 9**). The total global production, harvested area, and total import and export trade of coarse cereals have been on the rise in the past 10 years, demonstrating the gradually improving status of coarse cereals in world food security. Coarse cereals contain special functional components, such as flavonoids, linoleic acid, phenolic acid, and β-glucan. More importantly, due to their unique nature, different coarse cereal processing methods may affect or destroy these inherent functional components. In addition to the need to consider health aspects and delicious taste during processing, special processing processes or techniques, especially those that will retain functionally active ingredients, for example, should be the focus of future research. This will lead to a deeper consideration and utilization of coarse cereals to serve for human food security and healthy diets.

In this study, we recognize the advantages and development potential of coarse cereals in the face of today’s frequent extreme climate change (drought), but development of the coarse cereals industry still faces the problem of low and unstable resources. There is still a large gap in production compared to staple grains. Therefore, research on the production of coarse cereals is also imperative. Excellent germplasm resources are key to improving the yield and quality of mixed grains, and science- and technology-led efforts should be strengthened to cultivate more excellent germplasm resources. Considering the above, and analyzing the relationship between “environment–hybridization measures,” variety characteristics and regional characteristics, we can adjust and develop integrated-management strategies for hybrid agricultural production, and enhance the ability of these production systems to increase production, improve the sustainability of these systems, and increase the overall benefits of hybrid cultivation. For example, the more specific functional components (e.g., flavonoids), which are readily produced in coarse cereals in response to drought, can be considered rapid feedback indicators of adverse conditions; their formation has a synergistic relationship with the adverse environment as well as a defensive role under such an environment. Unfortunately, the synergistic relationship and the mechanisms of action are both unclear. Therefore, it is important to clarify these mechanisms for the construction of stress-resistant cultivation or high-quality cultivation management of coarse cereals, which may be an important way to enhance stress-resistance and quality of coarse cereals in the future.

**Figure 10:**
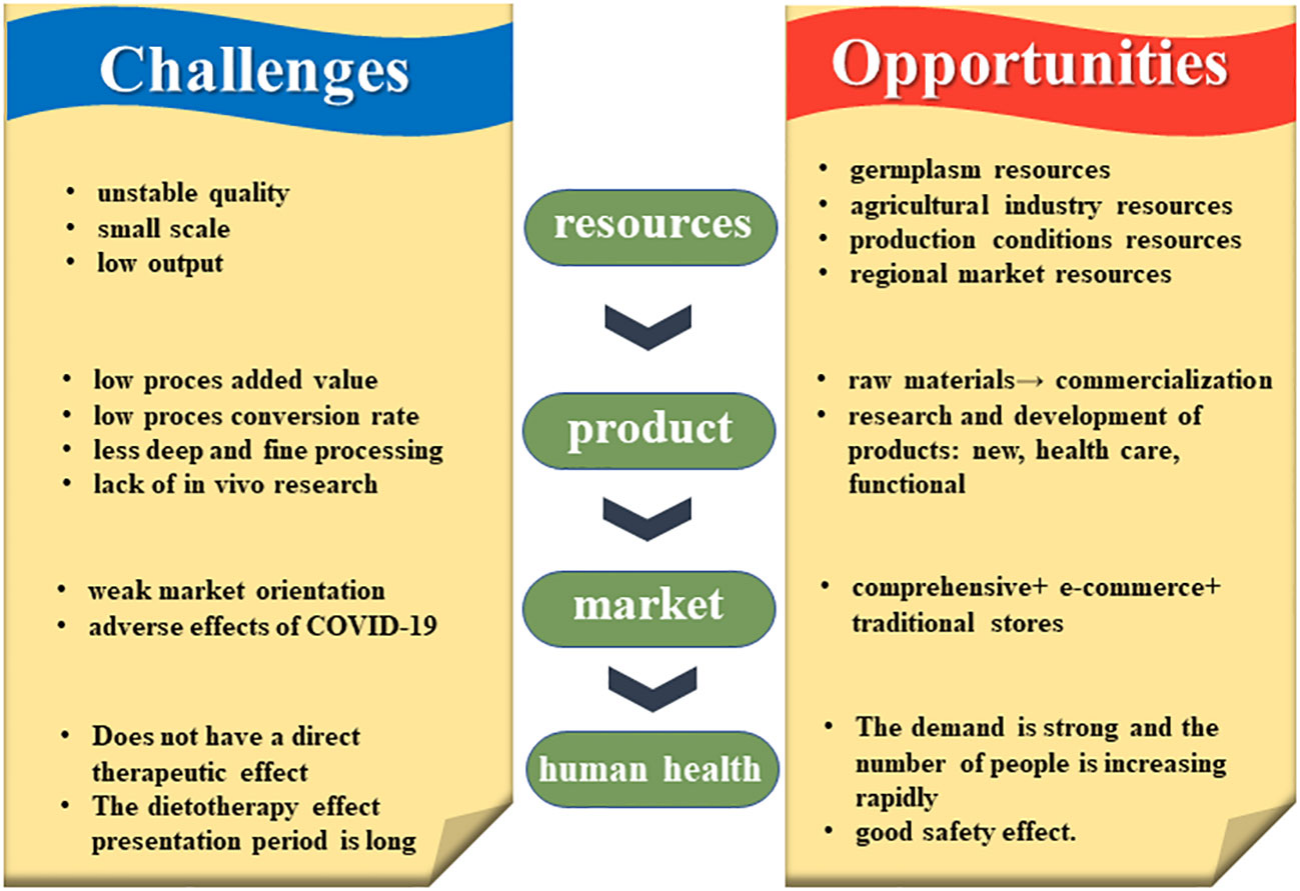
Challenges and opportunities faced by coarse cereals in world food security.

Taken together, the miscellaneous grains industry has great potential for development, but also faces many challenges, such as a lack of in-depth research and inefficient agricultural production systems (**Figure 10**). The former is needed for proper government guidance and the formulation of relevant policies, and the latter to help develop miscellaneous grains for the market, including production, processing, marketing, economic inputs, and industry chain extension. Development and strengthening of the miscellaneous grains industry will enable these grains to play a more important role in providing healthy human diets and world food security, among others.

## Author contributions

XZ: Writing – original draft. JZ: Writing – original draft. TC: Writing – original draft. YG: Writing – original draft. LZ: Writing – original draft. XH: Writing – original draft. CL: Writing – review & editing. YW: Writing – review & editing. XY: Writing – review & editing. XC: Writing – review & editing. CS: Writing – review & editing. GZ: Writing – review & editing. DX: Writing – review & editing.

## References

[B1] Abbas T. Fan R. Hussain S. Sattar A. Khalid S. Butt M.. (2022). Protective effect of jasmonic acid and potassium against cadmium stress in peas (Pisum sativum L.). Saudi J. Biol. Sci. 29, 2626–2633. doi: 10.1016/j.sjbs.2021.12.051 35531166 PMC9073065

[B2] Adamczewska-Sowinska K. Sowinski J. Jama-Rodzenska A. (2021). The effect of sowing date and harvest time on leafy greens of quinoa (chenopodium quinoa willd.) yield and selected nutritional parameters. Agriculture-Basel 11, 405. doi: 10.3390/agriculture11050405

[B3] Agnihotri A. Aruoma O. I. (2020). Alzheimer’s disease and Parkinson’s disease: A nutritional toxicology perspective of the impact of oxidative stress, mitochondrial dysfunction, nutrigenomics and environmental chemicals. J. Am. Coll. Nutr. 39 (1), 16–27. doi: 10.1080/07315724.2019.1683379 31829802

[B4] Akhtar M. S. Shakeel U. Siddiqui Z. A. (2010). Biocontrol of fusarium wilt by bacillus pumilus, pseudomonas alcaligenes, and rhizobium sp on lentil. Turkish J. Biol. 34, 1–7. doi: 10.3906/biy-0809-12

[B5] Akter S. Jahan I. Hossain M. A. Hossain M. A. (2021). Laboratory-and field-phenotyping for drought stress tolerance and diversity study in lentil (lens culinaris medik. ). Phyton-International J. Exp. Bot. 90, 949–970. doi: 10.32604/phyton.2021.014411

[B6] Ames N. Dreiseitl A. Steffenson B. J. Muehlbauer G. J. (2015). Mining wild barley for powdery mildew resistance. Plant Pathol. 64, 1396–1406. doi: 10.1111/ppa.12384

[B7] Amezqueta S. Galan E. Fuguet E. Carrascal M. Abian J. Torres J. (2012). Determination of d-fagomine in buckwheat and mulberry by cation exchange HPLC/ESI–Q-MS. Analytical Bioanalytical Chem. 402, 1953–1960. doi: 10.1007/s00216-011-5639-2 22207282

[B8] Anantharaju P. G. Gowda P. C. Manjunath G. V. Madhunapantula S. (2016). An overview on the role of dietary phenolics for the treatment of cancers. Nutr. J. 15. doi: 10.1186/s12937-016-0217-2 PMC5131407 27903278

[B9] Araya A. Prasad P. V. V. Ciampitti I. A. Jha P. K. (2021). Using crop simulation model to evaluate influence of water management practices and multiple cropping systems on crop yields: A case study for Ethiopian highlands. Field Crops Res. 260. doi: 10.1016/j.fcr.2020.108004

[B10] Atkinson C. J. Fitzgerald J. D. Hipps N. A. (2010). Potential mechanisms for achieving agricultural benefifits from biochar application to temperate soils: A review. Plant Soil 337, 1–18. doi: 10.1007/s11104-010-0464-5

[B11] Augé R. M. (2001). Water relations, drought and vesicular-arbuscular mycorrhizal symbiosis. Mycorrhiza 11, 3–42. doi: 10.1007/s005720100097

[B12] Food and Agriculture Organization of the United Nations (FAO). (2022). (FAOSTAT). Available at: https://www.fao.org/faostat/zh/#data/QCL (Accessed February 22, 2022).

[B13] Azpilicueta M. Irigoyen I. Lasa B. Muro J. Aparicio-Tejo P. M. (2012). Yield and quality of sugar snap pea in the Ebro Valley: Sowing date and seed density. Scientia Agricola 69, 320–326. doi: 10.1590/S0103-90162012000500006

[B14] Babarashi E. Rokhzadi A. Pasari B. Mohammadi K. (2021). Ameliorating effects of exogenous paclobutrazol and putrescine on mung bean vigna radiata (l.) wilczek under water deficit stress. Plant Soil Environ. 67, 40–45. doi: 10.17221/437/2020-PSE

[B15] Bazghaleh N. Prashar P. Woo S. Vandenberg A. (2020). Effects of lentil genotype on the colonization of beneficial trichoderma species and biocontrol of aphanomyces root rot. Microorganisms 8, 1290. doi: 10.3390/microorganisms8091290 32846963 PMC7564536

[B16] Benaffari W. Boutasknit A. Anli M. Ait-El-Mokhtar M. Ait-Rahou Y. Ben-Laouane R.. (2022). The native arbuscular mycorrhizal fungi and vermicompost-based organic amendments enhance soil fertility, growth performance, and the drought stress tolerance of quinoa. Plants-Basel 11, 393. doi: 10.3390/plants11030393 35161374 PMC8838481

[B17] Benevenuto J. Teixeira-Silva N. S. Kuramae E. E. Croll D. Monteiro-Vitorello C. B. (2018). Comparative genomics of smut pathogens: insights from orphans and positively selected genes into host specialization. Front. In Microbiol. 9. doi: 10.3389/fmicb.2018.00660 PMC5897528 29681893

[B18] Beniwal D. Dhall R. K. Yadav S. Sharma P. (2022). An overview of rust (uromyces viciae-fabae) and powdery mildew. Genetika-Belgrade 54, 499–512. doi: 10.2298/GENSR2201499B

[B19] Bernstein A. M. Titgemeier B. Kirkpatrick K. Golubic M. Roizen M. F. (2013). Major cereal grain fibers and psyllium in relation to cardiovascular health. Nutrients 5, 1471–1487. doi: 10.3390/nu5051471 23628720 PMC3708330

[B20] Bianco-Gomes A. C. Nogueira L. D. Bono-Lopes N. V. M. Gouvea-Souza C. P. Boldrini-Franca J. Gomes V. M.. (2022). Dry heat and pressure favor bioactive compounds preservation and peptides formation in sorghum sorghum bicolor (l. ) moench. Curr. Res. Food Sci. 5, 117–124. doi: 10.1016/j.crfs.2021.12.013 35036931 PMC8749381

[B21] Bilal T. Malik B. Tahir I. Rehman R. Hakeem K. R. Alharby H. (2019). Aluminium stress modulates the osmolytes and enzyme defense system in Fagopyrum species. Plant Physiol. Biochem. 144. doi: 10.1016/j.plaphy.2019.09.033 31574383

[B22] Bolan N. S. (1991). A critical review of the role of mycorrhizae fungi in the uptake of phosphorus by plants. Plant Soil 134, 189–207. doi: 10.1007/bf00012037

[B23] Borovaya S. A. Klykov A. G. (2020). Some aspects of flavonoid biosynthesis and accumulation in buckwheat plants. Plant Biotechnol. Rep. 14, 213–225. doi: 10.1007/s11816-020-00614-9

[B24] Bouis H. E. (2000). Enrichment of food staples through plant breeding: a new strategy for fighting micronutrient malnutrition. Nutrition 16, 701–704. doi: 10.1016/S0899-9007(00)00266-5 10906604

[B25] Canales F. J. Montilla-Bascon G. Rispail N. Prats E. (2019). Salicylic acid regulates polyamine biosynthesis during drought responses in oat. Plant Signaling Behav. 14, 1–4. doi: 10.1080/15592324.2019.1651183 PMC6768256 31382811

[B26] Castaldo L. Izzo L. Gaspari A. Lombardi S. Rodriguez-Carrasco Y. Narvaez A.. (2022). Chemical composition of green pea (pisum sativum l.) pods extracts and their potential exploitation as ingredients in nutraceutical formulations. Antioxidants 11, 105. doi: 10.3390/antiox11010105 PMC8772770 35052609

[B27] Chamanabad H. R. M. Ghorbani A. Asghari A. Tulikov A. M. Zargarzadeh F. (2009). Long-term effects of crop rotation and fertilizers on weed community in spring barley. Turkish J. Agric. Forestry 33, 315–323. doi: 10.3906/tar-0712-47

[B28] Chen C. Y. Milbury P. E. Kwak H. K. Collins F. W. Samuel P. Blumberg J. B. (2004). Avenenthramides and phenolic acids from oats are bioavailable and act synergistically with Vit C to enhance hamster and human LDL resistance to oxidation. J. Nutr. 134, 1459–1466. doi: 10.1093/jn/134.6.1459 15173412

[B29] Cheng W. G. Kimani S. M. Kanno T. Tang S. R. Oo A. Z. Tawaraya K.. (2018). Forage rice varieties Fukuhibiki and Tachisuzuka emit larger CH4 than edible rice Haenuki. Soil Sci. Plant Nutr. 64, 77–83. doi: 10.1080/00380768.2017.1378569

[B30] Choi J. Y. Lee J. M. Lee D. G. Cho S. Yoon Y. H. Cho E. J.. (2015). The n-butanol fraction and rutin from Tartary buckwheat improve cognition and memory in an in *vivo* model of amyloid-β-induced Alzheimer’s disease. J. Medicinal Food 18 (6), 631–641. doi: 10.1089/jmf.2014.3292 25785882

[B31] Ciudad-Mulero M. Barros L. Fernandes A. Berrios J. D. J. Cámara M. Morales P.. (2018). Bioactive compounds and antioxidant capacity of extruded snack-type products developed from novel formulations of lentil and nutritional yeast flours. Food Funct. 9, 819–829. doi: 10.1039/C7FO01730H 29359222

[B32] Colque-Little C. Abondano M. C. Lund O. S. Amby D. B. Piepho H. P. Andreasen C.. (2021). Genetic variation for tolerance to the downy mildew pathogen Peronospora variabilis in genetic resources of quinoa (Chenopodium quinoa). BMC Plant Biol. 21, 41. doi: 10.1186/s12870-020-02804-7 33446098 PMC7809748

[B33] Costa J. F. Cosio W. Cardenas M. Yabar E. Gianoli E. (2009). Preference of quinoa moth: eurysacca melanocampta meyrick (lepidoptera: gelechiidae) for two varieties of quinoa (chenopodium quinoa willd.) in olfactometry assays. Chilean J. Agric. Res. 69, 71–78. doi: 10.4067/S0718-58392009000100009

[B34] Coulibaly W. H. Bouatenin K. M. J. P. Boli Z. B. I. A. Alfred K. K. Bi Y. C. T. N’sa K. M. C.. (2020). Influence of yeasts on bioactive compounds content of traditional sorghum beer (tchapalo) produced in cote d’ivoire. Curr. Res. Food Sci. 3, 195–200. doi: 10.1016/j.crfs.2020.06.001 32914135 PMC7473363

[B35] del Hierro J. N. Reglero G. Martin D. (2020). Chemical characterization and bioaccessibility of bioactive compounds from saponin-rich extracts and their acid-hydrolysates obtained from fenugreek and quinoa. Foods 9, 1159. doi: 10.3390/foods9091159 32839396 PMC7555840

[B36] Delin S. Engstrom L. Lundkvist A. (2018). Optimal placement of meat bone meal pellets to spring oats. Front. In Sustain. Food Syst. 2. doi: 10.3389/fsufs.2018.00027

[B37] Deng R. Zhao H. Xiao Y. Huang Y. Yao P. Lei Y.. (2019). Cloning, characterization, and expression analysis of eight stress-related NAC genes in tartary buckwheat. Crop Sci. 59, 266–279. doi: 10.2135/cropsci2018.06.0368

[B38] Do T. D. T. Cozzolino D. Muhlhausler B. S. Box A. Able A. J. (2015). Antioxidant capacity and vitamin e in barley: Effect of genotype and storage. Food Chem. 187, 65–74. doi: 10.1016/j.foodchem.2015.04.028 25976999

[B39] Domingos I. Bilsborrow P. (2022). Optimizing quinoa growth cycle duration in northeast England by varying the sowing date. Agron. J. 114, 2186–2199. doi: 10.1002/agj2.21131

[B40] Du T. S. Kang S. Z. Zhang J. H. Davies W. J. (2015). Deficit irrigation and sustainable water-resource strategies in agriculture for China’s food security. J. Exp. Bot. 66, 2253–2269. doi: 10.1093/jxb/erv034 25873664 PMC4868834

[B41] Emmons C. L. Peterson D. M. Paul G. L. (1999). Antioxidant activity and contents of phenolic and toco1 antioxidant. J. Agr Food Chem. 47, 4894–4898. 10606549 10.1021/jf990530i

[B42] Erol S. Arslan O. Asik B. B. Carpici E. B. (2022). The effects of different sowing times and harvest stages on forage yield and quality in buckwheat (Fagopyrum esculentum Moench). Turkish J. Field Crops 27, 78–86. doi: 10.17557/tjfc.1071283

[B43] Escudero-Martinez C. Rodriguez P. A. Liu S. Santos P. A. Stephens J. Bos J. I. B. (2020). An aphid effector promotes barley susceptibility through suppression of defence gene expression. J. Exp. Bot. 71, 2796–2807. doi: 10.1093/jxb/eraa043 31989174 PMC7210766

[B44] FAO IFAD UNICEF WFP WHO (2022). The State of Food Security and Nutrition in the World 2022. Repurposing food and agricultural policies to make healthy diets more affordable (Rome: FAO). doi: 10.4060/cc0639en

[B45] Fazalova V. Nevado B. (2020). Low spontaneous mutation rate and pleistocene radiation of pea aphids. Mol. Biol. And Evol. 37, 2045–2051. doi: 10.1093/molbev/msaa066 32163142

[B46] Felderhoff T. J. McIntyre L. M. Saballos A. Vermerris W. (2016). Using genotyping by sequencing to map two novel anthracnose resistance loci in sorghum bicolor. G3-Genes Genomes Genet. 6, 1935–1946. doi: 10.1534/g3.116.030510 PMC4938647 27194807

[B47] Ferrazza J. M. Soares A. B. Martin T. N. Assmann A. L. Nicola V. (2013). Production of annual winter forages at different sowing times Produção de forrageiras anuais de inverno em diferentes épocas de semeadura. Rev. Ciencia Agronomica 44, 379–389. doi: 10.1590/S1806-66902013000200022

[B48] Fghire R. Anaya F. Ali O. I. Benlhabib O. Ragab R. Wahbi S. (2015). Physiological and photosynthetic response of quinoa to drought stress. Chilean J. Agric. Res. 75, 174–183. doi: 10.4067/S0718-58392015000200006

[B49] Figueiredo M. V. B. Burity H. A. Martinez C. Chanway C. P. (2008). Alleviation of drought stress in the common bean (Phaseolus vulgaris L.) by co-inoculation with Paenibacillus polymyxa and Rhizobium tropici. Appl. Soil Ecol. 40, 182–188. doi: 10.1016/j.apsoil.2008.04.005

[B50] Flower K. C. Cordingley N. Ward P. R. Weeks C. (2012). Nitrogen, weed management and economics with cover crops in conservation agriculture in a mediterranean climate. Field Crops Res. 132, 63–75. doi: 10.1016/j.fcr.2011.09.011

[B51] Fu J. Zhang Y. Hu Y. C. Zhao G. Tang Y. Zou L. (2020). Concise review: Coarse cereals exert multiple beneficial effects on human health. Food Chem. 325. doi: 10.1016/j.foodchem.2020.126761 32387947

[B52] Fujiwara Y. Shimada S. Takumi S. Murai K. (2010). Differential effects of Aegilops tauschii genotypes on maturing-time in synthetic hexaploid wheats. Breed. Sci. 60, 286–292. doi: 10.1270/jsbbs.60.286

[B53] Galgano F. Tolve R. Scarpa T. Caruso M. C. Lucini L. Senizza B.. (2021). Extraction kinetics of total polyphenols, flavonoids, and condensed tannins of lentil seed coat: Comparison of solvent and extraction methods. Foods 10, 1810. doi: 10.3390/foods10081810 34441587 PMC8393944

[B54] Gano B. Dembele J. S. B. Tovignan T. K. Sine B. Vadez V. Diouf D.. (2021). Adaptation responses to early drought stress of west africa sorghum varieties. Agronomy-Basel 11, 443. doi: 10.3390/agronomy11030443

[B55] Gao F. Yao H. Zhao H. Zhou J. Luo X. Huang Y.. (2016). Tartary buckwheat FtMYB10 encodes an R2R3-MYB transcription factor that acts as a novel negative regulator of salt and drought response in transgenic Arabidopsis. Plant Physiol. Biochem. 109, 387–396. doi: 10.1016/j.plaphy.2016.10.022 27814568

[B56] Gao W. Y. Zhang Y. J. Feng Z. Bai Q. Q. He J. J. Wang Y. J. (2018). Effects of melatonin on antioxidant capacity in naked oat seedlings under drought stress. Molecules 23, 1580. doi: 10.3390/molecules23071580 29966243 PMC6099629

[B57] Gao F. Zhou J. Deng R. Y. Zhao H. X. Li C. L. Chen H.. (2017). Overexpression of a tartary buckwheat R2R3-MYB transcription factor gene, FtMYB9, enhances tolerance to drought and salt stresses in transgenic Arabidopsis. J. Plant Physiol. 214, 81–90. doi: 10.1016/j.jplph.2017.04.007 28460279

[B58] Garmendia I. Rashidi S. Quezada-Salirrosas M. R. Goicoechea N. (2022). Atmospheric CO2 concentration affects the life cycle, yield, and fruit quality of early maturing edible legume cultivars. J. Sci. Food Agric. 102, 3964–3971. doi: 10.1002/jsfa.11743 34952971

[B59] Gol L. Haraldsson E. B. von Korff M. (2021). Ppd-H1 integrates drought stress signals to control spike development and flowering time in barley. J. Of Exp. Bot. 72 (1), 122–136. doi: 10.1093/jxb/eraa261 32459309 PMC7816852

[B60] Gong W. L. Ju Z. L. Chai J. K. Zhou X. R. Lin D. D. Su W. J.. (2022). Physiological and Transcription Analyses Reveal the Regulatory Mechanism in Oat (Avena sativa) Seedlings with Different Drought Resistance under PEG-Induced Drought Stress. Agronomy-Basel 12 (5). doi: 10.3390/agronomy12051005

[B61] Guenther J. F. Chanmanivone N. Galetovic M. P. Wallace I. Cobb J. A. Roberts D. M. (2003). Phosphorylation of soybean nodulin 26 on serine 262 enhances water permeability and is regulated developmentally and by osmotic signals. Plant Cell 15, 981–991. doi: 10.1105/tpc.009787 12671092 PMC152343

[B62] Guo W. M. Nie L. Wu D. Y. Wise M. L. Collins F. W. Meydani S. N.. (2010). Avenanthramides inhibit proliferation of human colon cancer cell lines in vitro. Nutr. Cancer-an Int. J. 62 (8), 1007–1016. doi: 10.1080/01635581.2010.492090 21058188

[B63] Hafez Y. Attia K. Alamery S. Ghazy A. Al-Doss A. Ibrahim E.. (2020). Beneficial effects of biochar and chitosan on antioxidative capacity, osmolytes accumulation, and anatomical characters of water-stressed barley plants. Agronomy-Basel 10, 630. doi: 10.3390/agronomy10050630

[B64] Hagels H. (2007). “Sekundare Pflanzeninhaltstofe des Buchweizen (secondary plant compounds of buckwheat: The effects of rutin, extraction of rutin from buckwheat leaves),” in Eds. Kreft I. Ries C. Zewen C.. Das Buchweizen Buch: mit Rezepten aus aller Welt. (Arzfeld, Luxemburg: Islek ohne Grenzen), 103–109.

[B65] Hakala K. Jauhiainen L. Rajala A. A. Jalli M. Kujala M. Laine A. (2020). Different responses to weather events may change the cultivation balance of spring barley and oats in the future. Field Crops Res. 259. doi: 10.1016/j.fcr.2020.107956

[B66] Haling R. E. Brown L. K. Bengough A. G. Young I. M. Hallett P. D. White P. J.. (2013). Root hairs improve root penetration, rootsoil contact, and phosphorus acquisition in soils of different strength. J. Exp. Bot. 64, 3711–3721. doi: 10.1093/jxb/ert200 23861547

[B67] Hao J. X. Li J. X. Zhao D. D. (2021). Effect of slightly acidic electrolysed water on functional components, antioxidant and alpha-glucosidase inhibitory ability of buckwheat sprouts. Int. J. Food Sci. Technol. 56, 3463–3473. doi: 10.1111/ijfs.14972

[B68] Hassan I. Basahi J. (2014). Effects of enhanced UV-B radiation and drought stress on photosynthetic performance of lettuce (Lactuca sativa L. Romaine) plants. Annu. Res. Rev. Biol. 4, 1739–1756. doi: 10.9734/ARRB/2014/6638

[B69] He H. Y. Hu Q. Li R. Pan X. B. Huang B. X. He Q. J. (2020). Regional gap in maize production, climate and resource utilization in China. Field Crops Res. 254. doi: 10.1016/j.fcr.2020.107830

[B70] He W. F. Ye F. M. Wang H. H. Zhao Q. Xu M. (2022). Phenotypic identification and diversity of vigna radiata L. Germplasm in liaoning, China. SSRN Electronic J. 14. doi: 10.2139/ssrn.4030293

[B71] Herrera T. del Hierro J. N. Fornari T. Reglero G. Martin D. (2019). Acid hydrolysis of saponin-rich extracts of quinoa, lentil, fenugreek and soybean to yield sapogenin-rich extracts and other bioactive compounds. J. Sci. Food Agric. 99, 3157–3167. doi: 10.1002/jsfa.9531 30536393

[B72] Hidri R. Barea J. M. Metoui-Ben Mahmoud O. Abdelly C. Azcon R. (2016). Impact of microbial inoculation on biomass accumulation by sulla carnosa provenances, and in regulating nutrition, physiological and antioxidant activities of this species under non-saline and saline conditions. J. Plant Physiol. 201, 28–41. doi: 10.1016/j.jplph.2016.06.013 27393918

[B73] Hinojosa L. Gonzalez J. A. Barrios-Masias F. H. Fuentes F. Murphy K. M. (2018). Quinoa abiotic stress responses: A review. Plants-Basel 7, 106. doi: 10.3390/plants7040106 30501077 PMC6313892

[B74] Holou R. A. Y. Stevens G. (2012). Juice, sugar, and bagasse response of sweet sorghum (Sorghum bicolor (L.) Moench cv. M81E) to N fertilization and soil type. Global Change Biol. Bioenergy 4, 302–310. doi: 10.1111/j.1757-1707.2011.01126.x

[B75] Hossain M. S. Li J. Sikdar A. Hasanuzzaman M. Uzizerimana F. Muhammad I.. (2020). Exogenous melatonin modulates the physiological and biochemical mechanisms of drought tolerance in tartary buckwheat (fagopyrum tataricum(l. ) gaertn). Molecules 25, 2828. doi: 10.3390/molecules25122828 32570970 PMC7355475

[B76] Hostetler G. Ralston R. A. Schwartz S. J. (2017). Flavones: food sources, bioavailability, metabolism, and bioactivity. Adv. Nutr. 8, 423–435. doi: 10.3945/an.116.012948 28507008 PMC5421117

[B77] Hou D. Z. Yousaf L. Xue Y. Hu J. R. Wu J. H. Hu X. S.. (2019). Mung bean (vigna radiata l. ): Bioactive polyphenols polysaccharides peptides Health benefits. Nutrients 11, 1238. doi: 10.3390/nu11061238 31159173 PMC6627095

[B78] Huan X. J. Li L. Liu Y. J. Kong Z. Y. Liu Y. J. Wang Q. C.. (2022). Integrating transcriptomics and metabolomics to analyze quinoa (Chenopodium quinoa Willd.) responses to drought stress and rewatering. Front. Plant Sci. 13. doi: 10.3389/fpls.2022.988861 PMC9645111 36388589

[B79] Huang J. Chen Q. J. Rong Y. P. Tang B. Zhu L. W. Ren R. R.. (2021). Transcriptome analysis revealed gene regulatory network involved in PEG-induced drought stress in Tartary buckwheat (Fagopyrum Tararicum). Peerj 9. doi: 10.7717/peerj.11136 PMC8019315 33850661

[B80] Huang J. Deng J. Shi T. Chen Q. Liang C. Meng Z.. (2017). Global transcriptome analysis and identification of genes involved in nutrients accumulation during seed development of rice tartary buckwheat (Fagopyrum Tararicum). Sci. Rep. 7, 11792. doi: 10.1038/s41598-017-11929-z 28924217 PMC5603606

[B81] Hussain F. Khan E. A. Baloch M. S. Aziz K. A. Khan Q. U. (2022). Impact of seasonal variability on phenological development and productivity of mungbean (vigna radiata (l.) R. Wilczek) in arid climatic condition. Appl. Ecol. Environ. Res. 20, 2985. doi: 10.15666/aeer/2004_29852999

[B82] Idehen E. Tang Y. Sang S. M. (2016). Bioactive phytochemicals in barley. J. Food Drug Anal. 25, 148–161. doi: 10.1016/j.jfda.2016.08.002 28911532 PMC9333424

[B83] Indiati S. W. Hapsari R. T. Prayogo Y. Sholihin Sundari T. Megaya M.. (2021). Resistance level of mung bean genotypes to pod borer maruca testulalis geyer. Legume Res. 44, 602–607. doi: 10.18805/LR-590

[B84] Ji L. L. Lay D. Chung E. Fu Y. Peterson D. M. (2003). Effect of avenathramides on oxidant generation and antioxidant enzyme activity in exercised rats. Nutr. Res. 23, 1579–1590. doi: 10.1016/S0271-5317(03)00165-9

[B85] Jiang G. H. Suhail M. Khalid M. Zhao C. (2021). Profiling and characterization of oat cultivars (avena sativa l.) with respect to bioactive compounds, pesticide residues and mycotoxin. Int. J. Food Properties 24, 1187–1201. doi: 10.1080/10942912.2021.1954658

[B86] Jones J. M. (2006). Grain-based foods and health cereals. Cereal Foods World 51, 108–113.

[B87] Jones P. J. H. Raeini-Sarjaz M. Ntanios F. Y. Vanstone C. A. Feng J. Y. Parsons W. E. (2000). Modulation of plasma lipid levels and cholesterol kinetics by phytosterol versus phytostanol esters. J. Lipid Res. 41, 697–705. doi: 10.1016/S0022-2275(20)32378-6 10787430

[B88] Jonsson M. Wratten S. D. Robinson K. A. Sam S. A. (2009). The impact of floral resources and omnivory on a four trophic level food web. Bull. Entomological Res. 99, 275–285. doi: 10.1017/S0007485308006275 19063752

[B89] Jordan-Meille L. Holland J. E. McGrath S. P. Glendining M. J. Thomas C. L. Haefele S. M. (2021). The grain mineral composition of barley, oat and wheat on soils with pH and soil phosphorus gradients. Eur. J. Agron. 126. doi: 10.1016/j.eja.2021.126281

[B90] Jung G. H. Kim S. L. Kim M. J. Kim S. K. Park J. H. Kim C. G.. (2015). Effect of sowing time on buckwheat (Fagopyrum esculentum Moench) growth and yield in central Korea. J. Crop Sci. Biotechnol. 18, 285–291. doi: 10.1007/s12892-015-0117-6

[B91] Kamali S. Mehraban A. (2020). Nitroxin and arbuscular mycorrhizal fungi alleviate negative effects of drought stress on sorghum bicolor yield through improving physiological and biochemical characteristics. Plant Signaling Behav. 15. doi: 10.1080/15592324.2020.1813998 PMC7588223 32902363

[B92] Kandel Y. R. Bradley C. A. Chilvers M. I. Mathew F. M. Tenuta A. U. Simth D. L.. (2019). Effect of seed treatment and foliar crop protection products on sudden death syndrome and yield of soybean. Plant Dis. 103, 1712–1720. doi: 10.1094/PDIS-12-18-2199-RE 31059383

[B93] Karagiannidis N. Hadjisavva-Zinoviadi S. (1998). The mycorrhizal fungus Glomus mosseae enhances the growth, yield and chemical composition of duram wheat in 10 different soils. Nutrient Cycling Agroecosystems 52, 1–7. doi: 10.1023/A:1016311118034

[B94] Katz D. L. Nawaz H. Boukhalil J. Chan W. Ahmadi R. Giannamore V.. (2001). Effects of oat and wheat cereals on endothelial responses. Prev. Med. 33, 476–484. 11676590 10.1006/pmed.2001.0918

[B95] Kaur K. D. Jha A. Sabikhi L. Singh A. K. (2014). Significance of coarse cereals in health and nutrition: A review. J. Food Sci. Technology-Mysore 51 (8), 1429–1441. doi: 10.1007/s13197-011-0612-9 PMC4108649 25114333

[B96] Kebede A. Z. Friesen-Enns J. Gnanesh B. N. Menzies J. G. Fetch J. W. M. Chong J.. (2019). Mapping oat crown rust resistance gene pc45 confirms association with PcKM. G3-Genes Genomes Genet. 9, 505–511. doi: 10.1534/g3.118.200757 PMC6385968 30554147

[B97] Keshtkar A. Aien A. Naghavi H. Najafinezhad H. Shirzadi M. H. (2022). Effect of the application of foliar jasmonic acid and drought stress on grain yield and some physiological and biochemical characteristics of chenopodium quinoa cultivars. J. Agric. Sciences-Tarim Bilimleri Dergisi 28, 171–180. doi: 10.15832/ankutbd.714568

[B98] Khalid W. Ali A. Arshad M. S. Afzal F. Akram R. Siddeeg A.. (2022). Nutrients and bioactive compounds of sorghum bicolor l. Used to prepare functional foods: A review on the efficacy against different chronic disorders. Int. J. Food Properties 25, 1045–1062. doi: 10.1080/10942912.2022.2071293

[B99] Khalid S. Rehman A. Mansha M. A. Mukhtar A. Rehman M. (2020). Exploring phyto-biochemical and nutraceutical profiling of buckwheat. J. Agric. Environ. Food Secur. 2, 32–73.

[B100] Khan M. S. Ahmad D. Khan M. A. (2015). Utilization of genes encoding osmoprotectants in transgenic plants for enhanced abiotic stress tolerance. Electronic J. Biotechnol. 18, 257–266. doi: 10.1016/j.ejbt.2015.04.002

[B101] Khan I. H. Javaid A. (2022). Hexane soluble bioactive components of leaf extract of quinoa. J. Anim. Plant Sciences-Japs 32, 609–614. doi: 10.36899/JAPS.2022.2.0461

[B102] Killi D. Haworth M. (2017). Diffusive and metabolic constraints to photosynthesis in quinoa during drought and salt stress. Plants-Basel 6, 49. doi: 10.3390/plants6040049 29039809 PMC5750625

[B103] Klajn V. M. Ames C. W. Cunha K. F. D. Lorini A. Hackbart H. C. D. S. Filho P. J. S.. (2021). Probiotic fermented oat dairy beverage: Viability of lactobacillus casei, fatty acid profile, phenolic compound content and acceptability. J. Food Sci. Technology-Mysore 58, 3444–3452. doi: 10.1007/s13197-021-04973-1 PMC8292467 34366461

[B104] Kothari K. Ale S. Bordovsky J. P. Porter D. O. Munster C. L. Hoogenboom G. (2020). Potential benefits of genotype-based adaptation strategies for grain sorghum production in the Texas High Plains under climate change. Eur. J. Agron. 117. doi: 10.1016/j.eja.2020.126037

[B105] Kurtz E. S. Wallo W. (2007). Colloidal oatmeal: history, chemistry and clinical properties. J. Drugs Dermatol. 6, 167–170. 17373175

[B106] Landete J. M. Hernandez T. Robredo S. Duenas M. Rivas B. D. L. Estrella I.. (2015). Effect of soaking and fermentation on content of phenolic compounds of soybean (glycine max cv. Merit) and mung beans (vigna radiata l wilczek). Int. J. Food Sci. Nutr. 66, 203–209. doi: 10.3109/09637486.2014.986068 25582183

[B107] Lehman R. M. Taheri W. I. Osborne S. L. Buyer J. S. Douds D. D. (2012). Fall cover cropping can increase arbuscular mycorrhizae in soils supporting intensive agricultural production. Appl. Soil Ecol. 61, 300–304. doi: 10.1016/j.apsoil.2011.11.008

[B108] Levidow L. Zaccaria D. Maia R. Vivas E. Todorovic M. Scardigno A. (2014). Improving water-efficient irrigation: Prospects and difficulties of innovative practices. Agric. Water Manage. 146, 84–94. doi: 10.1016/j.agwat.2014.07.012

[B109] Leybourne D. J. Valentine T. A. Binnie K. Taylor A. Karley A. J. Bos J. I. B. (2022). Drought stress increases the expression of barley defence genes with negative consequences for infesting cereal aphids. J. Exp. Bot. 73, 2238–2250. doi: 10.1093/jxb/erac010 35090009

[B110] Li Y. H. Lv P. Mi J. Z. Zhao B. P. Liu J. H. (2022). Integrative transcriptome and metabolome analyses of the interaction of oat-oat stem rust. Agronomy-Basel 12, 2353. doi: 10.3390/agronomy12102353

[B111] Li Q. Wu Q. Wang A. Lv B. Dong Q. Yao Y.. (2019). Tartary buckwheat transcription factor FtbZIP83 improves the drought/salt tolerance of Arabidopsis via an ABA-mediated pathway. Plant Physiol. Biochem. 144, 312–323. doi: 10.1016/j.plaphy.2019.10.003 31606716

[B112] Li Z. H. Zhao X. Y. Zhang X. W. Liu H. K. (2021). Bioactive compounds and biological activities of sorghum grains. Foods 10, 2868. doi: 10.3390/foods10112868 34829151 PMC8618165

[B113] Lim I. Kim B. C. Park Y. Park N. Ha J. (2022). Metabolic and developmental changes in germination process of mung bean (vigna radiata (l.) r. Wilczek) sprouts under different water spraying interval and duration. J. Food Qual., 1–13. doi: 10.1155/2022/6256310

[B114] Lin P. H. Chao Y. Y. (2021). Different drought-tolerant mechanisms in quinoa (chenopodium quinoa willd.) and djulis (chenopodium formosanum koidz.) based on physiological analysis. Plants-Basel 10, 2279. doi: 10.3390/plants10112279 34834642 PMC8620838

[B115] Liu A. Hamel C. Hamilton R. I. Ma B. L. Smith D. L. (2000). Acquisition of Cu, Zn, Mn, and Fe by mycorrhizal maize (Zea mays L.) grown in soil at different P and micronutrient levels. Mycorrhiza 9, 331–336. doi: 10.1007/s005720050277

[B116] Liu L. Zubik L. Collins F. W. Marko M. Meydani M. (2004). The antiatherogenic potential of oat phenolic compounds. Atherosclerosis 175, 39–49. doi: 10.1016/j.atherosclerosis.2004.01.044 15186945

[B117] Ma Y. L. Zhou S. M. Lu J. (2022). Metabolomic analysis reveals changes of bioactive compounds in mung beans (vigna radiata) during gamma-aminobutyric acid enrichment treatment. Foods 11, 1423. doi: 10.3390/foods11101423 35626988 PMC9141900

[B118] Maheshwari G. Sowrirajan S. Joseph B. (2019). β-Glucan, a dietary fiber in effective prevention of lifestyle diseases-An insight. Bioactive Carbohydrates Dietary Fibre 19. doi: 10.1016/j.bcdf.2019.100187

[B119] Malik A. H. Holm L. Johansson E. (2014). Governing plant development in barley (hordeum vulgare l.): Relation to protein composition and breakdown rates of protein polymers during malting. J. Sci. Food Agric. 94, 1559–1567. doi: 10.1002/jsfa.6457

[B120] Malik E. M. Muller C. E. (2016). Anthraquinones as pharmacological tools and drugs. Medicinal Res. Rev. 36, 705–748. doi: 10.1002/med.21391 27111664

[B121] Maphosa L. Anwar M. R. Luckett D. J. Ip R. H. L. Chauhan Y. S. Richards M. F. (2022). Impact of sowing time and genotype on water use efficiency of lentil (lens culinaris medick. ). Agronomy-Basel 12, 1542. doi: 10.3390/agronomy12071542

[B122] Marulanda A. Barea J. M. Azcon R. (2009). Stimulation of plant growth and drought tolerance by native microorganisms (am fungi and bacteria) from dry environments: Mechanisms related to bacterial effectiveness. J. Plant Growth Regul. 28, 115–124. doi: 10.1007/s00344-009-9079-6

[B123] McKee G. Cowger C. Dill-Macky R. Friskop A. Gautam P. Ransom J.. (2019). Disease management and estimated effects on don (deoxynivalenol) contamination in fusarium infested barley. Agriculture-Basel 9, 155. doi: 10.3390/agriculture9070155

[B124] Mehmood A. Naeem M. Khalid F. Saeed Y. Abbas T. Jabran K.. (2018). Identification of phytotoxins in different plant parts of Brassica napus and their influence on mung bean. Environ. Sci. pollut. Res. 25, 18071–18080. doi: 10.1007/s11356-018-2043-x 29691745

[B125] Meng H. L. Sun P. Y. Wang J. R. Sun X. Q. Zheng C. Z. Fan T.. (2022). Comparative physiological, transcriptomic, and WGCNA analyses reveal the key genes and regulatory pathways associated with drought tolerance in Tartary buckwheat. Front. Plant Sci. doi: 10.3389/fpls.2022.985088 PMC9575659 36262653

[B126] Menga V. Codianni P. Fares C. (2014). Agronomic management under organic farming may affect the bioactive compounds of lentil (lens culinaris l.) and grass pea (lathyrus communis l.)? Sustainability 6, 1059–1075. doi: 10.3390/su6021059

[B127] Metabolic (2017) The global food system: An analysis. Available at: https://www.metabolic.nl/publication/global-food-system-an-analysis/ (Accessed November 4, 2023).

[B128] Miransari M. (2014). Plant growth promoting rhizobacteria. J. Plant Nutr. 37, 2227–2235. doi: 10.1080/01904167.2014.920384

[B129] Mirosavljevic M. Przulj N. Momcilovic V. Hristov N. Maksimovic I. (2015). Dry matter accumulation and remobilization in winter barley as affected by genotype and sowing date. Genetika-Belgrade 47, 751–763. doi: 10.2298/GENSR1502751M

[B130] Mott J. Abaye O. Reiter M. Maguire R. (2022). Evaluating effects of bradyrhizobium and arbuscular mycorrhizal fungi inoculation on yield components of mung bean (vigna radiata (l.) wilczek) and nitrogen fixation. Agronomy-Basel 12, 2358. doi: 10.3390/agronomy12102358

[B131] Mwamahonje A. Eleblu J. S. Y. Ofori K. Feyissa T. Deshpande S. Garcia-Oliveira A. L.. (2021). Introgression of qtls for drought tolerance into farmers’ preferred sorghum varieties. Agriculture-Basel 11, 883. doi: 10.3390/agriculture11090883

[B132] Nahar K. Hasanuzzaman M. Alam M. M. Fujita M. (2015). Glutathione-induced drought stress tolerance in mung bean: Coordinated roles of the antioxidant defence and methylglyoxal detoxification systems. Aob Plants. doi: 10.1093/aobpla/plv069 PMC4526754 26134121

[B133] Nxele X. Klein A. Ndimba B. K. (2017). Drought and salinity stress alters ros accumulation, water retention, and osmolyte content in sorghum plants. South Afr. J. Bot. 108, 261–266. doi: 10.1016/j.sajb.2016.11.003

[B134] Olmo M. Lopez-Iglesias B. Villar R. (2014). Drought changes the structure and elemental composition of very fine roots in seedlings of ten woody tree species. Implications for a drier climate. Plant Soil 384, 113–129. doi: 10.1007/s11104-014-2178-6

[B135] O’Moore K. M. Vanlandschoot C. M. Dickman J. R. Figi A. R. Rothert A. M. Ji L. L. F. (2005). Effect of avenanthramides on rat skeletal muscle injury induced by lengthening contraction. Med. Sci. Sports Exer 37 (Suppl), S466.

[B136] Oten M. (2017). The effects of different sowing time and harvesting height on hydrocyanic acid content in some silage sorghum (sorghum bicolor l. ) varieties. Turkish J. Field Crops 22, 211–217. doi: 10.17557/tjfc.356224

[B137] Pala F. (2019). A survey on weed management in dry lentil fields. Appl. Ecol. Environ. Res. 17, 13513–13521. doi: 10.15666/aeer/1706_1351313521

[B138] Paucar-Menacho L. M. Martinez-Villaluenga C. Dueñas M. Frias J. Peñas E. (2018). Response surface optimisation of germination conditions to improve the accumulation of bioactive compounds and the antioxidant activity in quinoa. Int. J. Food Sci. Technol. 53, 516–524. doi: 10.1111/ijfs.13623

[B139] Piasecka A. Sawikowska A. Kuczynska A. Ogrodowicz P. Mikolajczak K. Krystkowiak K.. (2017). Drought-related secondary metabolites of barley (hordeum vulgare l.) leaves and their metabolomic quantitative trait loci. Plant J. 89, 898–913. doi: 10.1111/tpj.13430 27880018

[B140] Przybylska-Balcerek A. Frankowski J. Stuper-Szablewska K. (2020). The influence of weather conditions on bioactive compound content in sorghum grain. Eur. Food Res. Technol. 246, 13–22. doi: 10.1007/s00217-019-03391-0

[B141] Punnuri S. M. Ayele A. G. Harris-Shultz K. R. Knoll J. E. Coffin A. W. Tadesse H. K.. (2022). Genome-wide association mapping of resistance to the sorghum aphid in Sorghum bicolor. Genomics 114. doi: 10.1016/j.ygeno.2022.110408 35716823

[B142] Qiao G. Wen X. P. Yu L. F. Ji X. B. (2011). The enhancement of drought tolerance for pigeon pea inoculated by arbuscular mycorrhizae fungi. Plant Soil Environ. 57, 541–546. doi: 10.17221/116/2011-PSE

[B143] Rai K. N. Gowda C. L. L. Reddy B. V. S. Sehgal S. (2008). Adaptation and potential uses of sorghum and pearl millet in alternative and health foods. Compr. Rev. Food Sci. Food Saf. 7, 340–352. doi: 10.1111/j.1541-4337.2008.00049.x

[B144] Raymundo R. Sexton-Bowser S. Ciampitti I. A. Morris G. P. (2021). Crop modeling defines opportunities and challenges for drought escape, water capture, and yield increase using chilling-tolerant sorghum. Plant Direct 5, e349. doi: 10.1002/pld3.349 34532633 PMC8436229

[B145] Reddy G. V. P. Shrestha G. Miller D. A. Oehlschlager A. C. (2018). Pheromone-trap monitoring system for pea leaf weevil, sitona lineatus: effects of trap type, lure type and trap placement within fields. Insects 9, 75. doi: 10.3390/insects9030075 29954083 PMC6164004

[B146] Roohi E. Mohammadi R. Niane A. A. Niazian M. Niedbala G. (2022). Agronomic performance of rainfed barley genotypes under different tillage systems in highland areas of dryland conditions. Agronomy-Basel 12, 1070. doi: 10.3390/agronomy12051070

[B147] Roychoudhury A. Paul S. Basu S. (2013). Cross-talk between abscisic acid-dependent and abscisic acid-independent pathways during abiotic stress. Plant Cell Rep. 32, 985–1006. doi: 10.1007/s00299-013-1414-5 23508256

[B148] Ruan J. Zhou Y. Yan J. Zhou M. Woo S. H. Weng W.. (2020). Tartary buckwheat: An under-utilized edible and medicinal herb for food and nutritional security. Food Rev. Int. 38, 1–15. doi: 10.1080/87559129.2020.1734610

[B149] Saberi B. Golding J. B. Chockchaisawasdee S. Scarlett C. J. Stathopoulos C. E. (2018). Effect of biocomposite edible coatings based on pea starch and guar gum on nutritional quality of “valencia” orange during storage. Starch-Starke 70, 5–6. doi: 10.1002/star.201700299

[B150] Sadeghipour O. (2019). Polyamines protect mung bean vigna radiata (l.) wilczek plants against drought stress. Biol. Futura 70, 71–78. doi: 10.1556/019.70.2019.09 34554428

[B151] Salacheep S. Kasemsiri P. Pongsa U. Okhawilai M. Chindaprasirt P. Hiziroglu S. (2020). Optimization of ultrasound-assisted extraction of anthocyanins and bioactive compounds from butterfly pea petals using taguchi method and grey relational analysis. J. Food Sci. Technology-Mysore 57, 3720–3730. doi: 10.1007/s13197-020-04404-7 PMC7447736 32903992

[B152] Salehi A. Mehdi B. Fallah S. Kaul H. P. Neugschwandtner R. W. (2018). Productivity and nutrient use efficiency with integrated fertilization of buckwheat-fenugreek intercrops. Nutrient Cycling Agroecosystems 110, 407–425. doi: 10.1007/s10705-018-9906-x

[B153] Sallam A. Alqudah A. M. Dawood M. F. A. Baenziger P. S. Borner A. (2019). Drought stress tolerance in wheat and barley: Advances in physiology, breeding and genetics research. Int. J. Mol. Sci. 20. doi: 10.3390/ijms20133137.71 PMC6651786 31252573

[B154] Saltzman E. Das S. K. Lichtenstein A. H. Dallal G. E. Corrales A. Schaefer E.. (2001). An oat-containing hypocaloric diet reduces systolic blood pressure and improves lipid profile beyond effects of weight loss in men and women. J. Nutr. 131, 1465–1470. doi: 10.1093/jn/131.5.1465 11340101

[B155] Sarapat S. Songwattana P. Longtonglang A. Umnajkitikorn K. Girdthai T. Tittabutr P.. (2020). Effects of increased 1-aminocyclopropane-1-carboxylate (acc) deaminase activity in bradyrhizobium sp. Sutn9-2 on mung bean symbiosis under water deficit conditions. Microbes Environments 35. doi: 10.1264/jsme2.ME20024 PMC7511786 32554939

[B156] Sayed M. A. Schumann H. Pillen K. Naz A. A. Léon J. (2012). Ab-qtl analysis reveals new alleles associated to proline accumulation and leaf wilting under drought stress conditions in barley (hordeum vulgare l.). BMC Genet. 13, 61. doi: 10.1186/1471-2156-13-61 22817330 PMC3532146

[B157] Seleiman M. F. Al-Suhaibani N. Ali N. Akmal M. Alotaibi M. Refay Y.. (2021). Drought stress impacts on plants and different approaches to alleviate its adverse effects. Plants-Basel 10, 259. doi: 10.3390/plants10020259 33525688 PMC7911879

[B158] Shahwar D. Bhat T. M. Ansari M. Y. K. Chaudhary S. Aslam R. (2017). Health functional compounds of lentil (lens culinaris medik): A review. Int. J. Food Properties 20, S1–S15. doi: 10.1080/10942912.2017.1287192

[B159] Shen Y. M. Liu H. L. Chang S. T. Chao C. H. (2010). First report of anthracnose caused by colletotrichum acutatum on mung bean sprouts in Taiwan. Plant Dis. 94, 131–131. doi: 10.1094/PDIS-94-1-0131C 30754427

[B160] Shen Q. Peng X. X. He F. Li S. Q. Xiao Z. Y. Wang H. H.. (2021). First report of nigrospora osmanthi causing leaf spot on tartary buckwheat in China. Plant Dis. 105, 1227–1227. doi: 10.1094/PDIS-08-20-1773-PDN

[B161] Shi H. He X. Zhao Y. Lu S. Guo Z. (2020). Constitutive expression of a group 3 LEA protein from Medicago falcata (MfLEA3) increases cold and drought tolerance in transgenic tobacco. Plant Cell Rep. 39, 851–860. doi: 10.1007/s00299-020-02534-y 32240329

[B162] Shinozaki K. Yamaguchi-Shinozaki K. (2007). Gene networks involved in drought stress response and tolerance. J. Exp. Bot. 58, 221–227. doi: 10.1093/jxb/erl164 17075077

[B163] Silva D. D. Costa R. V. Cota L. V. Figueiredo J. E. F. Casela C. R. Lanza F. E. (2015). Genotype rotation for leaf anthracnose disease management in sorghum. Crop Prot. 67, 145–150. doi: 10.1016/j.cropro.2014.10.007

[B164] Singh M. Kumar S. Mehra R. Sood S. Malhotra N. Sinha R.. (2022). Evaluation and identification of advanced lentil interspecific derivatives resulted in the development of early maturing, high yielding, and disease-resistant cultivars under Indian agro-ecological conditions. Front. Plant Sci. 13. doi: 10.3389/fpls.2022.936572 PMC9499259 36161028

[B165] Singh B. U. Sharma H. C. Rao K. V. (2012). Mechanisms and genetic diversity for host plant resistance to spotted stem borer, Chilo partellus in sorghum, Sorghum bicolor. J. Appl. Entomology 135, 386–400. doi: 10.1111/j.1439-0418.2011.01647.x

[B166] Singh O. Singh D. K. Singh A. Singh R. P. Pandey S. Bajpai A. K. (2022). Varieties under alluvial soil of uttar pradesh by cluster front line demonstrations. Legume Res. 45, 492–496. doi: 10.18805/LR-4704

[B167] Song Y. J. Jarvis D. I. Bai K. Y. Feng J. C. Long C. L. (2020). Assessment of the resilience of a tartary buckwheat (Fagopyrum tataricum) cultivation system in meigu, southwest China. Sustainability 12, 5683. doi: 10.3390/su12145683

[B168] Song C. Xiang D. B. Yan L. Song Y. Zhao G. Wang Y. H.. (2016). Changes in Seed growth, levels and distribution of flavonoids during tartary buckwheat seed development. Plant Production Sci. 19, 518–527. doi: 10.1080/1343943X.2016.1207485

[B169] Staroske N. Conrad U. Kumlehn J. Hensel G. Radchuk R. Erban A.. (2016). Increasing abscisic acid levels by immunomodulation in barley grains induces precocious maturation without changing grain composition. J. Exp. Bot. 67, 2675–2687. doi: 10.1093/jxb/erw102 26951372 PMC4861016

[B170] Sterna V. Zute S. Jansone I. Kantane I. (2017). Chemical composition of covered and naked spring barley varieties and their potential for food production. Polish J. Food Nutr. Sci. 67, 151–158. doi: 10.1515/pjfns-2016-0019

[B171] Sun W. Ma Z. Chen H. Liu M. (2019). MYB gene family in potato (Solanum tuberosum L.): genome-wide identification of hormone-responsive reveals their potential functions in growth and development. Int. J. Mol. Sci. 20 (19), 4847. doi: 10.3390/ijms20194847 31569557 PMC6801432

[B172] Sytar O. Biel W. Smetanska I. Brestic M. (2018). “Bioactive compounds and their biofunctional properties of different buckwheat germplasms for food processing,” in Buckwheat Germplasm in the World. Eds. Zhou M. L. Kreft I. Suvorova G. Tang Y. Woo S. H. (Cambridge, MA, USA: Academic Press), 191–204.

[B173] Tan Q. H. Liu Y. J. Dai L. Pan T. (2021). Shortened key growth periods of soybean observed in China under climate change. Sci. Rep. 11, 8197. doi: 10.1038/s41598-021-87618-9 33854171 PMC8047036

[B174] Tan M. Temel S. (2018). Performance of some quinoa (chenopodium quinoa willd.) genotypes grown in different climate conditions. Terkish J. Field Crops 23, 180–186. doi: 10.17557/tjfc.485617

[B175] Tariq R. Hussain A. Tariq A. Khalid M. H. B. Khan I. Basim H.. (2022). Genome-wide analyses of the mung bean NAC gene family reveals orthologs, co-expression networking and expression profiling under abiotic and biotic stresses. BMC Plant Biol. 22, 343. doi: 10.1186/s12870-022-03716-4 35836131 PMC9284730

[B176] Tekin S. Yazar A. Barut H. (2017). Comparison of wheat-based rotation systems and monocropping systems under dryland Mediterranean conditions. Int. J. Agric. Biol. Eng. 10, 203–213. doi: 10.25165/j.ijabe.20171005.3443

[B177] Thomas S. C. Frye S. Gale N. Garmon M. Launchbury R. MaChado N.. (2013). Biochar mitigates negative effects of salt additions on two herbaceous plant species. J. Environ. Manage. 129, 62–68. doi: 10.1016/j.jenvman.2013.05.057 23796889

[B178] Tripolskaja L. Booth C. A. Fullen M. (2013). A lysimeter study of organic carbon leaching from green manure and straw into a sandy loam Haplic Luvisol. Zemdirbyste-Agriculture 100, 3–8. doi: 10.13080/z-a.2013.100.001

[B179] Turmel M. S. Entz M. H. Tenuta M. May W. E. LaFond G. P. (2011). The influence of a long-term black medic (medicago lupulina cv. George) cover crop on arbuscular mycorrhizal fungal colonization and nutrient uptake in flax (linum usitatissimum) under zero-tillage management. Can. J. Plant Sci. 91, 1071–1076. doi: 10.4141/CJPS10115

[B180] Umina P. A. Reidy-Crofts J. Babineau M. Maino J. L. Edwards O. R. (2020). Susceptibility of the bird cherry-oat aphid, Rhopalosiphum padi (Hemiptera: Aphididae), to four insecticides. Austral Entomology 59, 838–844. doi: 10.1111/aen.12490

[B181] Villacrés E. Quelal M. B. Galarza S. Iza D. Silva E. (2022). Nutritional value and bioactive compounds of leaves and grains from quinoa (chenopodium quinoa willd.). Plants-Basel 11. doi: 10.3390/plants11020213 PMC8777597 35050101

[B182] Wang Y. F. Pan F. B. Wang G. S. Zhang G. D. Wang Y. L. Chen X. S.. (2014). Effects of biochar on photosynthesis and antioxidative system of malus hupehensis rehd. Seedlings under replant conditions. Scientia Hortic. 175, 9–15. doi: 10.1016/j.scienta.2014.05.029

[B183] Wang Y. Zhang Y. Li Z. Z. Zhao Q. Huang X. Y. Huang K. F. (2019). Effect of continuous cropping on the rhizosphere soil and growth of common buckwheat. Plant Prod. Sci. 23, 81–90. doi: 10.1080/1343943X.2019.1685895

[B184] Wei M. J. Tang M. Y. Wang L. Y. Cheng X. X. Wu Y. W. Ouyang J. (2021). Endogenous bioactive compounds of naked oats (avena nuda l.) inhibit alpha-amylase and alpha-glucosidase activity. Lwt-Food Sci. Technol. 149, 111902. doi: 10.1016/j.lwt.2021.111902

[B185] Woo S. L. Filippis F. D. Zotti M. Vandenberg A. Hucl P. Bonanomi G. (2022). Pea-wheat rotation affects soil microbiota diversity, community structure, and soilborne pathogens. Microorganisms 10, 370. doi: 10.3390/microorganisms10020370 35208825 PMC8876268

[B186] Wu X. H. Zhang Y. He P. Y. Huang X. Y. Huang K. F. (2020). Effects of tillage methods on senescence and grain filling characteristics of Tartary buckwheat. Zemdirbyste-Agriculture 107, 301–308. doi: 10.13080/z-a.2020.107.038

[B187] Xiang D. B. Ma C. R. Song Y. Wu Q. Wu X. Y. Sun Y. X.. (2019a). Post-anthesis photosynthetic properties provide insights into yield potential of tartary buckwheat cultivars. Agronomy 9, 149. doi: 10.3390/agronomy9030149

[B188] Xiang D. B. Song Y. Wu Q. Ma C. R. Zhao J. L. Wan Y.. (2019b). Relationship between stem characteristics and lodging resistance of Tartary buckwheat (Fagopyrum tataricum). Plant Production Sci. 22, 201–210. doi: 10.1080/1343943X.2019.1577143

[B189] Xiang D. B. Wei W. Ouyang J. Y. Le L. Q. Zhao G. Peng L. X.. (2020). Nitrogen alleviates seedling stage drought stress response on growth and yield of tartary buckwheat. Int. J. Agric. Biol. 24, 1167–1177. doi: 10.17957/IJAB/15.1546

[B190] Xiang D. B. Zhao G. Wan Y. Tan M. L. Song C. Song Y. (2016). Effect of planting density on lodging-related morphology, lodging rate, and yield of tartary buckwheat (Fagopyrum tataricum). Plant Production Sci. 19, 479–488. doi: 10.1080/1343943X.2016.1188320

[B191] Xue Y. S. Teng Y. X. Chen M. H. Li Z. Wang G. R. (2021). Antioxidant activity and mechanism of avenanthramides: Double H^+^/e^-^ processes and role of the catechol, guaiacyl, and carboxyl groups. J. Agric. Food Chem. 69 (25), 7178–7189. doi: 10.1021/acs.jafc.1c01591 34156855

[B192] Xue Z. H. Wang C. Zhai J. Yu L. W. C. Chang H. R. Kou X. H.. (2016). Bioactive compounds and antioxidant activity of mung bean (vigna radiata l.), soybean (glycine max l.) and black bean (phaseolus vulgaris l.) during the germination process. Czech J. Food Sci. 34, 68–78. doi: 10.17221/434/2015-CJFS

[B193] Yamamoto E. Karakaya H. C. Knap H. T. (2000). Molecular characterization of two soybean homologs of Arabidopsis thaliana CLAVATA1 from the wild type and fasciation mutant. Biochim. Biophys. Acta 1491, 333–340. doi: 10.1016/S0167-4781(00)00061-0 10760600

[B194] Yang A. Z. Akhtar S. S. Li L. Fu Q. Li Q. F. Naeem M. A.. (2020). Biochar mitigates combined effects of drought and salinity stress in quinoa. Agronomy-Basel 10, 912. doi: 10.3390/agronomy10060912

[B195] Yao P. F. Li C. L. Zhao X. R. Li M. F. Zhao H. X. Guo J. Y.. (2017). Overexpression of a tartary buckwheat gene, ftbhlh3, enhances drought/oxidative stress tolerance in transgenic arabidopsis. Front. Plant Sci. 8. doi: 10.3389/fpls.2017.00625 PMC5403918 28487715

[B196] Zafar-ul-Hye M. Akbar M. N. Iftikhar Y. Abbas R. M. Zahid A. Fahad S.. (2021). Rhizobacteria inoculation and caffeic acid alleviated drought stress in lentil plants. Sustainability 13, 9603. doi: 10.3390/su13179603

[B197] Zahedi S. M. Karimi M. Venditti A. (2021). Plants adapted to arid areas: Specialized metabolites. Natural Product Res. 35, 3314–3331. doi: 10.1080/14786419.2019.1689500 31766886

[B198] Zhang S. Y. Ai J. Guo Y. N. Bai Y. Yao H. Wang F. G. (2023). Cloning and expression analysis of VrNAC13 gene in mung bean. Open Life Sci. 18 (1). doi: 10.1515/biol-2022-0627 PMC10329274 37426623

[B199] Zhao D. Wright D. L. Marois J. J. Mackowiak C. L. Brennan M. (2010). Improved growth and nutrient status of an oat cover crop in sod-based versus conventional peanut-cotton rotations. Agron. Sustain. Dev. 30, 497–504. doi: 10.1051/agro/2009045

[B200] Zheng Y. C. Jin X. Y. Wang J. Y. Chen W. Yang Z. Chen Y. ,. X.. (2023). SbNAC9 improves drought tolerance by enhancing scavenging ability of reactive oxygen species and activating stress-responsive genes of sorghum. Int. J. Mol. Sci. 24 (3). doi: 10.3390/ijms24032401 PMC9917103 36768724

[B201] Zhu X. L. Wang B. Q. Wang X. Wei X. H. (2022). Identification of the CIPK-CBL family gene and functional characterization of CqCIPK14 gene under drought stress in quinoa. BMC Genomics 23 (1). 10.1186/s12864-022-08683-6 PMC9204864 35710332

[B202] Zini P. B. Poletto T. Fantinel V. S. Jacques R. J. S. Nunes U. R. Muniz M. D. B.. (2022). Buckwheat seed quality and pathogenicity of Fusarium spp. in plants. J. Seed Sci. 44, e202244004. doi: 10.1590/2317-1545v44256994

[B203] Zou L. Wu D. T. Ren G. X. Hu Y. C. Peng L. X. Zhao J. L.. (2021). Bioactive compounds, health benefits, and industrial applications of Tartary buckwheat (Fagopyrum tataricum). Crit. Rev. Food Sci. Nutr. doi: 10.1080/10408398.2021.1952161 34278850

